# Trophectoderm-like cells from EPS cells enable generating EPS cell-derived post-implantation embryoids that complete gastrulation

**DOI:** 10.1093/procel/pwaf059

**Published:** 2025-08-11

**Authors:** Xuyang Wang, Ruoqi Cheng, Chenyang Wu, Haiyin Liu, Zining Li, Yunfei Huo, Bo Li, Dongyu Zhao, Cheng Li, Hongkui Deng, Jun Xu

**Affiliations:** Department of Cell Biology, School of Basic Medical Sciences, Peking University Stem Cell Research Center, Peking University Health Science Center, Peking University, Beijing 100191, China; School of Life Sciences, Center for Bioinformatics, Center for Statistical Science, Peking University, Beijing 100871, China; Department of Biomedical Informatics, School of Basic Medical Sciences, Peking University, Beijing 100191, China; State Key Laboratory of Vascular Homeostasis and Remodeling, Peking University, Beijing 100191, China; Department of Cell Biology, School of Basic Medical Sciences, Peking University Stem Cell Research Center, Peking University Health Science Center, Peking University, Beijing 100191, China; MOE Key Laboratory of Cell Proliferation and Differentiation, School of Life Sciences and MOE Engineering Research Center of Regenerative Medicine, School of Basic Medical Sciences, State Key Laboratory of Natural and Biomimetic Drugs, Peking University Health Science Center, Peking–Tsinghua Center for Life Sciences, Peking University, Beijing 100871, China; MOE Key Laboratory of Cell Proliferation and Differentiation, School of Life Sciences and MOE Engineering Research Center of Regenerative Medicine, School of Basic Medical Sciences, State Key Laboratory of Natural and Biomimetic Drugs, Peking University Health Science Center, Peking–Tsinghua Center for Life Sciences, Peking University, Beijing 100871, China; MOE Key Laboratory of Cell Proliferation and Differentiation, School of Life Sciences and MOE Engineering Research Center of Regenerative Medicine, School of Basic Medical Sciences, State Key Laboratory of Natural and Biomimetic Drugs, Peking University Health Science Center, Peking–Tsinghua Center for Life Sciences, Peking University, Beijing 100871, China; Department of Biomedical Informatics, School of Basic Medical Sciences, Peking University, Beijing 100191, China; State Key Laboratory of Vascular Homeostasis and Remodeling, Peking University, Beijing 100191, China; School of Life Sciences, Center for Bioinformatics, Center for Statistical Science, Peking University, Beijing 100871, China; MOE Key Laboratory of Cell Proliferation and Differentiation, School of Life Sciences and MOE Engineering Research Center of Regenerative Medicine, School of Basic Medical Sciences, State Key Laboratory of Natural and Biomimetic Drugs, Peking University Health Science Center, Peking–Tsinghua Center for Life Sciences, Peking University, Beijing 100871, China; Changping Laboratory, Beijing 102206, China; Department of Cell Biology, School of Basic Medical Sciences, Peking University Stem Cell Research Center, Peking University Health Science Center, Peking University, Beijing 100191, China

**Keywords:** extended pluripotent stem cells, trophectoderm, embryoids, gastrulation

## Abstract

Mouse extended pluripotent stem (EPS) cells have demonstrated significant potential for generating embryo models *in vitro*. However, their limited capacity for extraembryonic trophoblast development has hindered their use in constructing whole embryo models, particularly post-implantation embryoids. Here, we establish a stepwise induction protocol to generate trophectoderm-like cells from mouse EPS cells. These cells retain trophectoderm-specific transcriptomic features and can differentiate into trophoblast lineages *in vivo*. Moreover, combining these trophectoderm-like cells with EPS cell-derived primitive endoderm/epiblast bilineage structures enabled the robust generation of post-implantation embryoids in a transgene-free manner. EPS-derived embryoids recapitulate key developmental events of post-implantation mouse embryos, including the formation of the pro-amniotic cavity, anterior-posterior axis, primitive streak, gastrulation, and complex extraembryonic tissues. Notably, single-cell transcriptomic analysis revealed a high degree of transcriptional similarity between EPS-derived embryoids at day 6 and natural E7.5 mouse embryos. Our study presents a novel platform for modeling post-implantation mouse embryogenesis *in vitro*.

## Introduction

The development of synthetic embryo models using early stem cells has provided powerful platforms for exploring mammalian development *in vitro* (Ai et al., 2023; Bao et al., 2022b; Cornwall-Scoones and Zernicka-Goetz, 2021; Liu et al., 2023b; Metzger et al., 2018; Shahbazi et al., 2019; Shao and Fu, 2020; Xiang et al., 2020; Yu et al., 2021, 2023; Zhai et al., 2022). By using early stem cells, pre-implantation blastoids and post-implantation embryo-like structures have been successfully generated (Amadei et al., 2021; Bao et al., 2022a; Dupont et al., 2023; Girgin et al., 2021; Harrison et al., 2017; Langkabel et al., 2021; Li et al., 2019, 2023, 2024; Peng et al., 2025; Sozen et al., 2018; Wu et al., 2023a, 2023b; Xu et al., 2022; Yu et al., 2021; Zhang et al., 2019, 2023). Remarkably, these efforts have led to the creation of synthetic embryos at the post-gastrulation stage, capable of initiating early organogenesis and forming complex embryonic and extraembryonic compartments, similar to those of natural E8.5 mouse embryos (Amadei et al., 2022; Lau et al., 2022; Tarazi et al., 2022). However, generating whole embryo-like entities in mice still relies on the substitution of extraembryonic stem cells with embryonic stem cells that transiently express master regulators of extraembryonic lineages, a process distinct from the natural specification of extraembryonic lineages *in vivo*. Due to the variability and restricted developmental potential of currently established extraembryonic stem cells (Amadei et al., 2021; Moerkamp et al., 2013; Paca et al., 2012), generating transgene-free post-implantation mouse embryo models capable of completing gastrulation *in vitro* remains a significant challenge.

One alternative strategy for generating embryo models is to use pluripotent stem cells with expanded developmental potentials. Unlike conventional pluripotent stem cells, extended pluripotent stem cells (EPS cells) or expanded potential stem cells can give rise to both embryonic and extraembryonic lineages (Gao et al., 2019; Liu et al., 2021; Xu et al., 2019; Yang et al., 2017b, 2017a; Yoshimatsu et al., 2023; Zheng et al., 2021). Notably, recent studies have demonstrated that EPS cells can be induced to form embryo-like structures, such as blastoids and peri-gastruloids (Li et al., 2019; Liu et al., 2023b; Luo and Yu, 2024; Min et al., 2022; Sozen et al., 2019, 2021; Xie et al., 2025; Zhang et al., 2022), highlighting their potential for modeling embryo development *in vitro*. However, a major limitation in utilizing EPS cells for embryo modeling is their restricted ability to generate extraembryonic trophectoderm (TE) lineages. Although EPS cells can efficiently generate extraembryonic primitive endoderm (PrE)-like cells that support the birth of live fetuses (Liu et al., 2023a), their TE-like derivatives still differ substantially from natural TE cells (Liu et al., 2023a; Posfai et al., 2021). This limitation has significantly hindered their use in generating high-quality post-implantation embryo models.

A promising strategy for generating high-quality TE lineages from mouse EPS cells is to develop a chemical approach that manipulates the signaling networks governing this process. The specification of TE lineages during early embryo development relies on the precise orchestration of diverse signaling pathways, such as FGF, WNT, TGFβ, and HIPPO (Dietrich et al., 2022; Nishioka et al., 2009; Seong et al., 2022; Tanaka et al., 1998; Wang et al., 2019). Importantly, the activities of these signaling pathways can be precisely fine-tuned *in vitro* in a spatio-temporal manner using small molecules, a strategy that has been shown to induce cellular fate changes across different lineages and stem cell types (Guan et al., 2022; Hou et al., 2013; Li et al., 2015; Liuyang et al., 2023; Wang et al., 2023). In principle, this approach could enhance the extraembryonic TE potency of EPS cells, a possibility that has yet to be explored.

In this study, we aimed to develop a chemical approach to generate high-quality TE lineages from mouse EPS cells. By sequentially modulating the signaling pathways of WNT, TGFβ, HIPPO, FGF, BMP, and PKA, we successfully induced TE-like cells (TELCs) from mouse EPS cells. Notably, the assembly of these TELCs with EPS-derived PrE/epiblast (EPI) bilineage structures enabled the efficient generation of post-implantation embryo-like structures, which progressed through gastrulation and developed into E7.5-like embryos *in vitro*.

## Results

### Identification of a chemical cocktail that supports TE fate induction from mouse EPS cells

**Figure 1.**
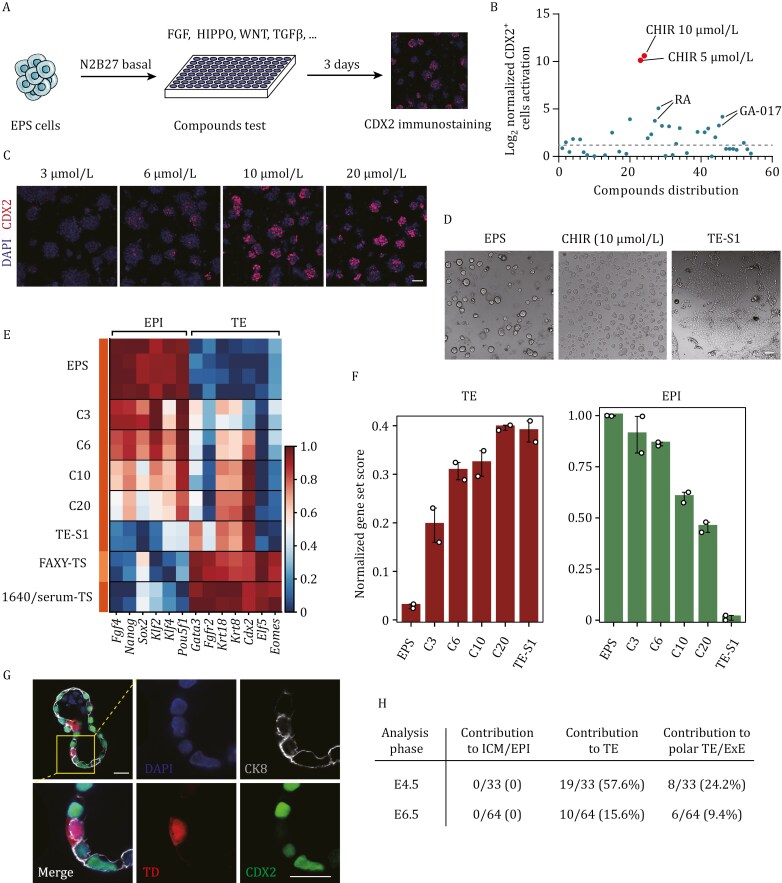
**Identification of a chemical cocktail enabling trophectoderm fate induction from mouse EPS cells.** (A) Schematic showing the strategy for identifying compounds capable of inducing the TE marker CDX2 in mouse EPS cells. (B) Results of compounds screening. The threshold was set to two-fold the normalized CDX2^+^ cell activation level relative to the DMSO control (dashed line). Values were normalized based on log_2_. Representative hit molecules are highlighted. CHIR, CHIR 99021. The compounds information and test results of all the tests are shown in Table S1. (C) Representative immunofluorescence images of CDX2 (red) in cells treated with 3, 6, 10, and 20 µmol/L CHIR 99021 for 3 days. Nuclei were counterstained with DAPI (blue). Scale bar, 50 µm. (D) Bright-field images depicting cellular morphology under various conditions. CHIR (10 µmol/L), EPS cells treated with 10 µmol/L CHIR 99021 for 3 days. TE-S1, EPS cells treated with TE-S1 medium for 3 days. Scale bar, 100 µm. (E) Heatmap showing the relative expression of representative epiblast (EPI) and trophectoderm (TE) genes in cells under different treatment conditions. C3/C6/C10/C20, EPS cells treated with 3/6/10/20 µmol/L CHIR 99021 for 3 days, respectively. TE-S1, EPS cells treated with TE-S1 medium for 3 days. FAXY-TS, TSCs cultured under FAXY-TSC medium (serum-free). 1640/serum-TS, TSCs cultured under 1640/serum-TSC medium (serum-containing). (F) Gene Set Variation Analysis (GSVA) enrichment scores for EPI and TE gene sets in cells under different treatment conditions. C3/C6/C10/C20, EPS cells treated with 3/6/10/20 µmol/L CHIR 99021 for 3 days, respectively. TE-S1, EPS cells treated with TE-S1 medium for 3 days. The gene lists are included in Table S3. (G) Representative immunofluorescent analysis showing expression of trophectoderm markers CDX2 (green) and CK8 (gray) in the chimeric blastocyst. Nuclei were counterstained with DAPI (blue). The yellow box highlights the magnified region displaying tdTomato (TD) positive cells. Scale bar, 25 µm. (H) Table summarizing outcomes of chimeric blastocyst and E6.5 embryos assays for cells treated with TE-S1 medium for 3 days. TD, tdTomato.

To induce TELCs from mouse EPS cells, we focused on modulating key signaling pathways involved in early embryo development, such as FGF, WNT, HIPPO, and TGFβ (Azami et al., 2019; Gu et al., 1998; Guzman-Ayala et al., 2004; Nishioka et al., 2009; Seong et al., 2022; Tanaka et al., 1998). To this end, we tested nearly 30 small molecules, cytokines, and growth factors for their ability to activate CDX2 expression (Fig. 1A and Table S1), a master regulator of TE fate in mice (Blij et al., 2015; Niwa et al., 2005; Strumpf et al., 2005). Notably, CHIR 99021, a GSK inhibitor that activates the WNT signaling pathway, induced the most significant activation of CDX2 expression in a dose-dependent manner (Fig. 1B and 1C). As the controls, FGF4 and bFGF, which are known to support the self-renewal of conventional trophoblast stem (TS) cells (Erlebacher et al., 2004; Kubaczka et al., 2014; Ohinata and Tsukiyama, 2014; Tanaka et al., 1998), had no effect on CDX2 activation in mouse EPS cells (Fig. S1A). Q-PCR analysis further showed that a high concentration of CHIR 99021 (10–20 μmol/L) was necessary to activate the endogenous TE markers *Cdx2* and *Krt8* to levels comparable to those in TS cells (Fig. S1B). However, activation of other representative TE marker genes, such as *Gata3* and *Id*2**, was not observed (Fig. S1C). Moreover, pluripotency markers were not significantly downregulated by CHIR 99021 treatment (Fig. S1C), suggesting that the pluripotency program was still maintained in these cells. Consistent with this, colonies treated with a high concentration of CHIR 99021 retained a dome-shaped morphology, similar to that of the original EPS colonies (Fig. 1D). These observations were further supported by bulk RNA-sequencing data (Fig. S1D–E), indicating that treatment with CHIR 99021 alone, at high concentration, is insufficient to induce complete TE differentiation from mouse EPS cells.

To further enhance the conversion of EPS cells to TELCs, we explored the combination of CHIR 99021 with compounds that regulate other signaling pathways. Through testing various chemical combinations, we identified a new cocktail consisting of CHIR 99021, GA-017, A8301, and FGF4 (TS-S1 condition), which efficiently induced mouse EPS cells to form flat epithelial colonies resembling conventional mouse trophoblast (TS) cells (Fig. 1D). In line with the morphological changes, Q-PCR and bulk RNA-sequencing analyses revealed significant downregulation of representative pluripotency marker genes, such as *Oct4*, *Nanog*, and *Klf4* (Figs. 1E and S1C). Consistent with these results, immunofluorescent analysis also showed the absent expression of NANOG in these cells (Fig. S1F). Moreover, Gene Set Variation Analysis (GSVA) showed a marked reduction in the gene set enriched in mouse EPS cells (Fig. 1F). Meanwhile, we observed a notable upregulation of several TE marker genes and TE-specific gene set in the converted cells (Figs. 1E, 1F and S1G). However, the key transcription factors *Eomes* and *Elf5*, which are typically expressed in E4.5 TE, remained lowly expressed in the converted cells (Fig. 1E).

Next, we conducted chimeric experiments to assess the *in vivo* developmental potential of the converted cells. To this end, tdTomato-labeled converted cells were injected into mouse 8-cell embryos, which were then cultured for two days in KSOM medium before the analysis. Notably, more than 20% of the analyzed embryos contained tdTomato-labeled cells, which were predominantly located in the mural TE region (Fig. 1G and 1H). Immunofluorescent analysis further revealed that these chimeric cells expressed CK8 but not CDX2 (Fig. 1G). We also attempted to transfer the injected 8-cell embryos into pseudo-pregnant mice, but none of the recovered E6.5 embryos (0/43) contained tdTomato-labeled cells (Fig. 1H). These results indicate that, despite successfully inducing TE fate from EPS cells, the converted cells fail to stably maintain the TE identity. Accordingly, these converted cells are referred to as pre-TELCs.

### A stepwise induction protocol enables the establishment of stable TELCs from mouse EPS cells

**Figure 2.**
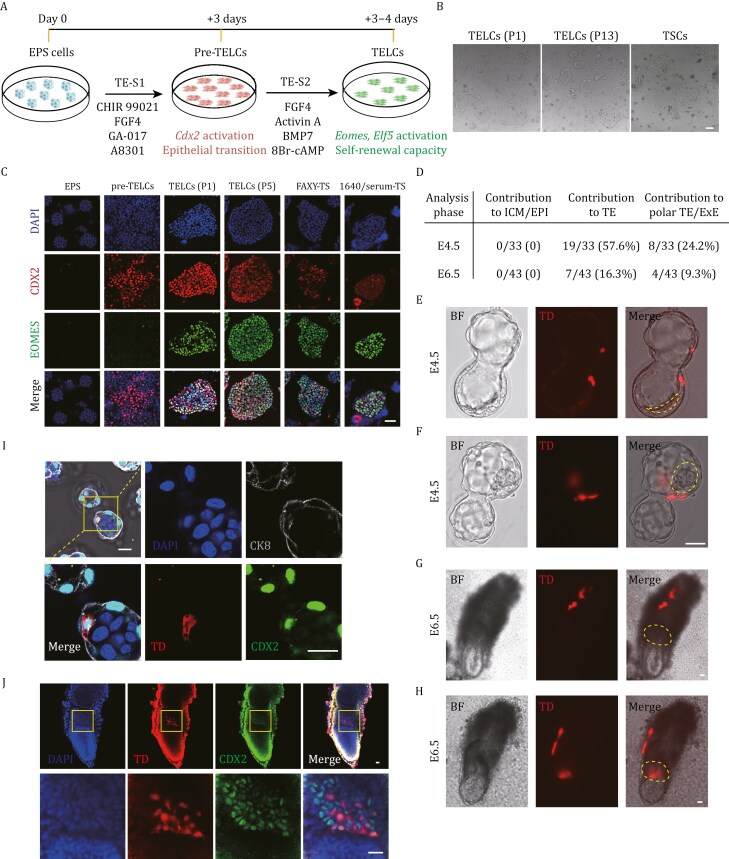
**Stepwise induction strategy enables the derivation of functional TELCs from mouse EPS cells.** (A) Schematic diagram of the stepwise induction protocol for TELCs derivation from mouse EPS cells. First stage: induction of EPS cells into pre-TELCs with TE-S1 medium. Second stage: maturation of pre-TELCs into TELCs in TE-S2 medium. (B) Bright-field images depicting cellular morphology under various conditions. TELCs (P1/13), TELCs cultured under TE-S2 medium for 1 or 13 passages, respectively. TSCs: TSCs cultured in serum-free FAXY-TSC medium. Scale bar, 100 µm. (C) Immunofluorescence staining of the trophectoderm markers CDX2 (red) and EOMES (green) in cell under various conditions. Nuclei were counterstained with DAPI (blue). TELCs (P1/5), TELCs cultured in TE-S2 medium for 1 or 5 passages, respectively. FAXY-TS, TSCs cultured in FAXY-TS medium. 1640/serum-TS, TSCs cultured in 1640/serum-TS medium. Scale bar, 25 µm. (D) Table summarizing outcomes of chimeric blastocyst and E6.5 embryos assays of TELCs. (E and F) Representative images showing the contribution of tdTomato (TD) labeled TELCs to the (E) mural TE; and (F) polar TE of the E4.5 blastocysts. Yellow dashed lines indicate the boundaries of the inner cell mass (ICM). Scale bar, 25 µm. (G and H) Representative images showing the contribution of tdTomato (TD) labeled EPS-TELCs to the (G) EPC; (H) ExE and TGCs of the E6.5 embryos. Yellow dashed outlines highlight extraembryonic ectoderm (ExE) region. Scale bar, 25 µm. (I) Representative immunofluorescent analysis showing expression of trophectoderm markers CDX2 (green) and CK8 (gray) in the chimeric blastocyst. Nuclei were counterstained with DAPI (blue). Yellow box highlights the magnified region displaying tdTomato (TD) positive cells. Scale bar, 25 µm. (J) Representative immunofluorescent analysis showing expression of ExE marker CDX2 (green) in the E6.5 chimeric embryo. Nuclei were counterstained with DAPI (blue). Yellow boxes indicate the ExE region of magnified inset showing CDX2-positive cells. Scale bar, 25 µm.

The chemical cocktail used to induce TE fate conversion included the WNT signaling agonist CHIR 99021 and the TGFβ inhibitor A8301, both of which have been shown to disrupt the self-renewal of mouse TS cells and promote trophoblast differentiation (Dietrich et al., 2022; Gu et al., 1998; Kubaczka et al., 2014; Ohinata and Tsukiyama, 2014). Based on this, we reasoned that prolonged exposure to this chemical cocktail could be detrimental to maintaining TE identity in pre-TELCs. Therefore, we sought to develop a stepwise induction protocol, comprising an initial induction of TE fate at stage 1, followed by the maintenance of the TE gene regulatory network at stage 2 (Fig. 2A). To achieve this, pre-TELCs were tested with various compound combinations targeting signaling pathways known to regulate the proliferation and self-renewal of TE cells (Azami et al., 2019; Erlebacher et al., 2004; Gardner et al., 1973; Gu et al., 1998; Kubaczka et al., 2014; Ohinata and Tsukiyama, 2014; Seong et al., 2022; Tanaka et al., 1998). This led to the identification of a novel cocktail containing FGF4, Activin A, BMP7, and 8r-cAMP (TE-S2 condition) (Fig. 2A), which supported the propagation of TS-like colonies from pre-TELCs (Fig. 2B). Immunofluorescence analysis further revealed that NANOG expression was completely absent in all cells treated under the TE-S2 condition, while only a few sporadic OCT4-positive cells were detected (Fig. S2A).

Next, we performed Q-PCR and immunofluorescent analyses to characterize the primary colonies at stage 2, as well as their long-term passaged progeny. Notably, Q-PCR analysis revealed robust activation of *Eomes* and *Elf5*, along with the sustained expression of other representative E4.5 TE marker genes (Fig. S2B). Additionally, we observed that the expression level of *Cdx2* in these cells was significantly higher when compared to conventional TS cells (Fig. S2B). Consistent with these findings, immunofluorescent analysis revealed strong expression of CDX2, EOMES, TFAP2C, GATA3, and CK8 in these cells (Figs. 2C and S2C–E). Importantly, the key pluripotency regulator OCT4 was absent in the primary colonies at stage 2 (Fig. S2E), suggesting a complete exit from the pluripotent state.

Given that previous studies have reported that TE-like cells from EPS cell-derived blastoids retain transcriptional features associated with PrE or embryonic mesoderm (Liu et al., 2023a; Posfai et al., 2021), we conducted immunofluorescence analysis to assess the expression of SOX17 and T, which are canonical markers for PrE and embryonic mesoderm, respectively. Notably, TELCs showed no detectable expression of either SOX17 or T (Fig. S2F). This observation was further supported by transcriptomic analysis, which revealed that, in contrast to TE-like cells from EPS-derived blastoids described in prior work (Liu et al., 2023a), TELCs lack expression of key marker genes associated with embryonic mesoderm and PrE (Fig. S2G). Additionally, TELCs did not exhibit enrichment of gene expression signatures characteristic of the PrE lineage (Fig. S2H).

To investigate the transcriptional dynamics during TELC generation, we identified gene sets that are relatively enriched in EPS cells, pre-TELCs, and TELCs, respectively (Fig. S2I). In addition, we analyzed gene expression differences between early- and late-passage TELCs and identified distinct gene sets associated with each stage (Fig. S2I). Subsequently, we performed GO enrichment analysis (Fig. S2I and Table S2). The initial EPS cells were enriched for GO terms associated with stem cell maintenance and DNA replication. In contrast, pre-TELCs exhibited enrichment for signaling pathways, including WNT and PI3K. Notably, GO terms related to FGF signaling were enriched in both early- and late-passage TELCs. Additionally, late-passage TELCs showed significant enrichment for GO terms linked to mitochondrial function (Fig. S2I and Table S2).

We further assessed the *in vivo* developmental potential of EPS-derived TELCs through chimeric experiments. Analysis of E4.5 embryos revealed that over 50% of the analyzed embryos (57.6%) contained tdTomato-labeled TELC derivatives in the TE region (Fig. 2D), which were located in either the mural or polar regions (Fig. 2E and 2F). More importantly, analysis of E6.5 embryos showed that 16.3% of chimeric embryos contained tdTomato-labeled cells in the ectoplacental cone (EPC), layer of trophoblast giant cells, or extraembryonic ectoderm (ExE) (Fig. 2G and 2H), all of which are derivatives of E4.5 TE. Given that Cdx2^+^ cells in the polar TE and ExE regions represent extraembryonic trophoblast stem or progenitor cells (Gardner et al., 1973; Hadas et al., 2024; Molè et al., 2020; Simmons and Cross, 2005; Strumpf et al., 2005), we performed immunofluorescent analysis using the E4.5 and E6.5 chimeric embryos. Notably, expression of CDX2 in the tdTomato-labeled cells was detected in these regions (Fig. 2I and 2J), suggesting that the TELC derivatives in the chimeric embryos still contain self-renewing extraembryonic trophoblast stem or progenitor cells. Collectively, these data indicate that mouse EPS cells can be efficiently induced into stable TELCs by stepwise modulation of signaling pathway combinations.

### Mouse EPS cell-derived TELCs at early passages maintain a pre-implantation E4.5 TE-like transcriptomic feature

**Figure 3.**
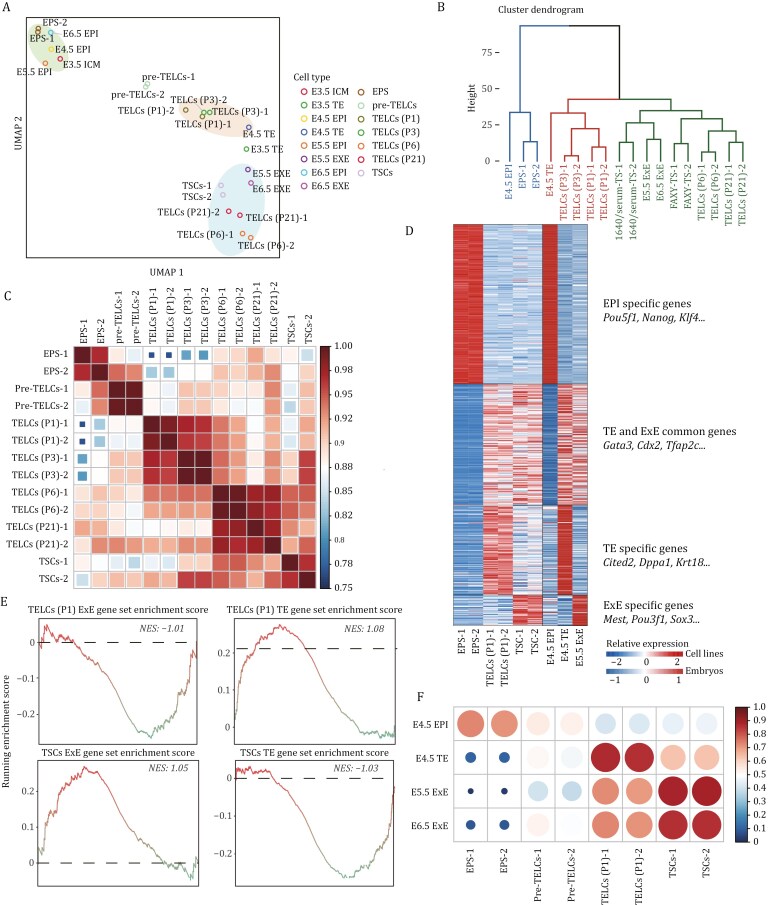
**Transcriptomic analysis reveals E4.5 TE-like features in early-passage TELCs.** (A) UMAP of bulk transcriptomes from mouse EPS cells, conventional TSCs, pre-TELCs (TE-S1), and EPS-derived TELCs cultured under TE-S2 medium at passages 1, 3, 6, and 21, integrated with published scRNA-seq data of epiblast and trophoblast lineages from pre-implantation to post-implantation stages (GSE123046). Points for scRNA-seq types show the median coordinate. See Methods for details. (B) Hierarchical clustering analysis of mouse EPS cells, conventional TSCs, and EPS-derived TELCs cultured under TE-S2 culture conditions at passages 1, 3, 6, and 21, as well as natural embryo samples: E4.5 EPI, E4.5 TE, and E5.5/E6.5 ExE, based on defined gene sets. (C) Pearson correlation matrix of global transcriptomes across mouse EPS cells, FAXY-TS cells, pre-TELCs (TE-S1), and EPS-derived TELCs cultured under TE-S2 culture conditions at passages 1, 3, 6, and 21. (D) Heatmap displaying the cell-state-specific gene expression in mouse EPS cells, conventional 1640/serum-TSCs and primary TELCs, as well as their corresponding mouse embryos lineages. The top color bar labels *in vitro* cell lines, and the bottom one corresponds to data from mouse embryos. (E) Gene set enrichment analysis (GSEA) of E4.5 TE and E5.5 ExE specific gene sets of primary TELCs and conventional FAXY-TS cells. (F) Pearson correlation analysis of mouse EPS cells, conventional FAXY-TS cells, pre-TELCs (TE-S1), and primary TELCs, based on E4.5 EPI, E4.5 TE, and E5.5 ExE gene sets.

To investigate the differentiation trajectory during TELCs induction, we conducted Uniform Manifold Approximation and Projection (UMAP) analysis to examine the global transcriptomes of different cell types involved in this study, including initial EPS cells, pre-TELCs, primary TELCs, TELCs from various passages, and conventional TS cells (Fig. 3A). To assess the transcriptomic similarities between *in vitro* cells and their *in vivo* counterparts, we also incorporated epiblast and trophoblast lineages from pre-implantation to post-implantation stages into the analysis. As a control, the initial EPS cells clustered alongside *in vivo* epiblast lineages (Fig. 3A). Notably, primary TELCs and their early-passage progeny were positioned close to the E4.5 TE, whereas pre-TELCs were located between EPS cells and TELCs (Fig. 3A). With prolonged passaging, TELCs at later passages progressively resembled post-implantation ExE and conventional TS cells (Fig. 3A). These findings were further supported by Principal Components Analysis (PCA), hierarchical clustering and Pearson correlation analyses (Figs. 3B, 3C and S2J).

Since the global transcriptome of TELCs at early passages differed significantly from that of conventional TS cells (Fig. 3A–C), we further investigated the transcriptional similarities and differences between these two cell types. Heatmap analysis revealed that genes enriched in the E4.5 epiblast were rarely expressed in early-passage TELCs or conventional TS cells (Fig. 3D). Moreover, both cell types expressed a core gene set associated with TS cell identity, which was also shared by E4.5 TE and E5.5 ExE (Fig. 3D). Additionally, we analyzed the expression of gene sets specific to E4.5 TE and E6.5 ExE in these cells. Importantly, E4.5 TE-enriched gene sets were expressed at significantly higher levels in primary TELCs compared to conventional TS cells (Fig. 3D), while the expression of the E6.5 ExE-related gene set was notably higher in conventional TS cells (Fig. 3D). The transcriptional similarity between primary TELCs and E4.5 TE was further confirmed by Gene Set Enrichment Analysis (GSEA) (Fig. 3E). To quantify the transcriptional similarities between *in vitro* cells and their *in vivo* counterparts, we performed Pearson correlation analysis. Among the various *in vitro* cell types, primary TELCs showed the highest correlation with E4.5 TE (Fig. 3F), whereas conventional TS cells exhibited a stronger resemblance to E5.5–E6.5 ExE (Fig. 3F). Collectively, these findings suggest that primary TELCs retain a pre-implantation, E4.5 TE-like transcriptomic signature, which is distinct from that of conventional TS cells.

### Efficient induction of PrE/EPI bilineage structures from mouse EPS cells

**Figure 4.**
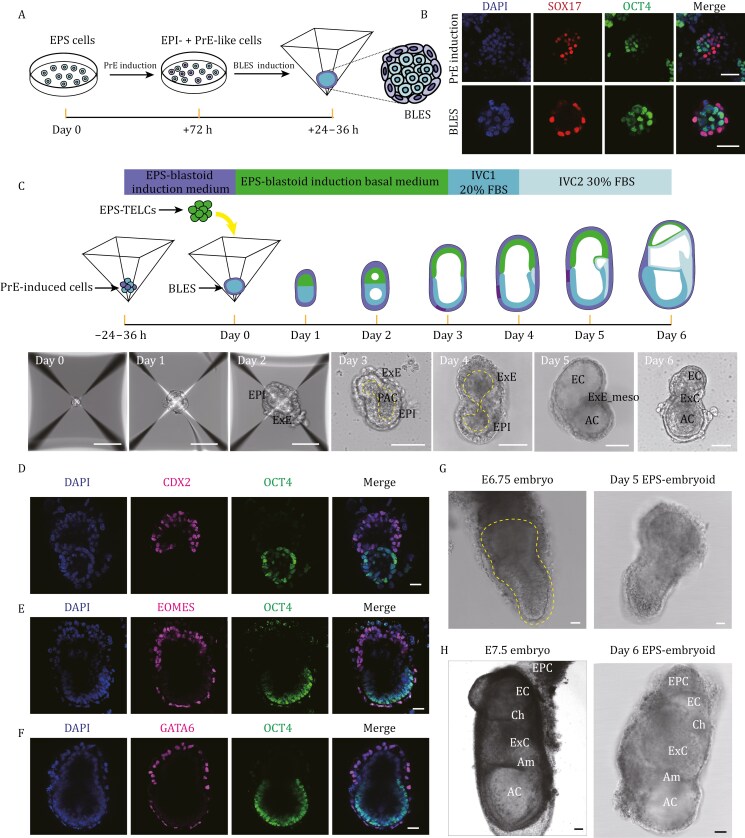
**EPS cell-derived embryoids mimic essential post-implantation morphological changes up to E7.*5.*** (A) Schematic diagram of the protocol for deriving PrE/EPI bilineage structures (BLES) from mouse EPS cells. Mouse EPS cells were firstly pretreated in PrE induction medium on Matrigel-coated plates, and then these cells were transferred to Aggrewell plates to form cell aggregates. (B) Representative immunofluorescent analysis showing the expression of SOX17 (red) and OCT4 (green) in (top) cells treated with PrE induction medium or (bottom) PrE/EPI bilineage structures (BLES). Nuclei were counterstained with DAPI (blue). Scale bar, 50 µm. (C) (Top) Schematic of EPS-embryoids generation. (Bottom) Representative bright-filed images of embryo-like structures from Day 0 to Day 6. Structures resembling the early post-implantation mouse embryo can be seen on Day 2. Yellow dashed outlines indicate the boundaries of pro-amniotic cavity (PAC)-like regions. PrE induced cells, cells treated with PrE induction medium for 3 days. BLES, PrE/EPI bilineage structures; EPI, epiblast; ExE, extraembryonic ectoderm; AC, amniotic cavity; EC, ectoplacental cavity; ExE_meso, extraembryonic mesoderm; ExC, exocoelomic cavity. Scale bar, 100 µm. (D–F) Representative immunofluorescent analysis showing the expression of epiblast marker (OCT4), extraembryonic ectoderm markers (CDX2, EOMES), visceral endoderm marker (GATA6) in Day 3 EPS-embryoids. Scale bar, 25 µm. (G) Representative bright-field images of (left) E6.75 natural mouse embryo and (right) Day 5 EPS-embryoid. Yellow dashed outline indicates the epiblast and extraembryonic ectoderm regions of the natural embryo. Scale bar, 25 µm. (H) Representative bright-field images of (left) E7.5 natural mouse embryo and (right) Day 6 EPS-embryoid. Am, amnion; AC, amniotic cavity; EC, ectoplacental cavity; ExC, exocoelomic cavity; Ch, chorion; EPC, ectoplacental cone. Scale bar, 50 µm.

The successful induction of primary TELCs from mouse EPS cells raises the question of whether these TELCs could be utilized to construct synthetic embryos. Given that mouse EPS cells can form PrE/EPI bilineage structures (Li et al., 2019; Liu et al., 2023a; Sozen et al., 2019), we hypothesized that these cells could be used to simultaneously generate EPI, PrE, and TE lineages, which could then be assembled to create transgene-free embryo models at the post-implantation stage. To explore this possibility, we first attempted to induce PrE-like cells from mouse EPS cells (Fig. 4A). By optimizing a previously reported PrE induction medium (FGF4, CHIR, 8Br-cAMP, and RA) (Vrij et al., 2022), we successfully induced cells expressing key PrE regulators within 3 days (Figs. S3A and 4B). Chimeric analysis further demonstrated that derivatives from these induced cells integrated into the EPI and visceral endoderm (VE) regions of E6.5 mouse embryos (Fig. S3B–C), suggesting the presence of both PrE-like and pluripotent cells following PrE induction.

Next, we utilized the mixture of PrE-like and pluripotent cells to induce PrE/EPI bilineage 3D structures (Fig. 4A). To this end, EPS cells treated with the PrE induction medium were transferred to AggreWell plates and further cultured in the EPS-blastoid medium (Li et al., 2019; Liu et al., 2023a). After 24–36 h of culture in the AggreWell plates, nearly all cell aggregates formed bilayer structures (Fig. S3D). Immunofluorescent analysis revealed that cell sorting occurred in all bilayer structures, with OCT4^+^ clusters surrounded by GATA6^+^ and SOX17^+^ cells (Figs. 4B and S3E).

We further performed bulk RNA sequencing to compare the transcriptomic features of the PrE/EPI bilineage structures, PrE-like/pluripotent mixtures, and the initial EPS cells. Compared to EPS cells, both the PrE/EPI bilineage structures and the PrE-like/pluripotent mixtures exhibited upregulation of PrE marker genes and downregulation of pluripotency marker genes (Fig. S3F and S3G), with the latter effect being more pronounced in the PrE/EPI bilineage structures (Fig. S3G). Consistent with these findings, PCA revealed distinct global transcriptional profiles between the PrE/EPI bilineage structures and the PrE-like/pluripotent mixtures (Fig. S3H). Heatmap analysis further showed increased expression of marker genes for PrE, parietal endoderm (ParE), and visceral endoderm (VE) in the PrE/EPI bilineage structures (Fig. S3I). Additionally, we observed upregulation of primed pluripotency marker genes accompanied by the downregulation of naive pluripotency marker genes in these structures (Fig. S3I). Collectively, these results demonstrate that PrE/EPI bilineage structures can be efficiently induced from mouse EPS cells, which are primed for the further development of epiblast and extraembryonic endoderm lineages at post-implantation stages.

### Egg-cylinder-shaped embryoids self-assembled solely from mouse EPS cells

Building on the successful generation of TELCs and PrE/EPI bilineage structures from mouse EPS cells, we attempted to investigate whether these EPS-derivatives could be assembled into embryo-like structures. To test this possibility, we first applied a previously established blastoid induction protocol to aggregates of TELCs and cells treated with PrE induction medium (Li et al., 2019; Liu et al., 2023a) (Fig. S4A). Within 48 h, blastocyst-like structures emerged, characterized by a tdTomato-positive TELC-derived cystic outer layer and an *Oct4*-GFP-positive inner cell mass-like region (Fig. S4B and S4C). Upon *in vivo* transplantation, these blastoids triggered decidualization, implying initial implantation competence (Fig. S4D). However, only degenerated structures were observed within the decidua (data not shown).

In addition to the developmental potential of the initiating stem cells, the blastoid induction environment is also known to significantly influence developmental outcomes (Li et al., 2019; Peng et al., 2025; Rivron et al., 2018; Sozen et al., 2019; Vrij et al., 2022; Xu et al., 2022; Zhang et al., 2023). By contrast, post-implantation embryoids can be generated robustly from co-aggregates of embryonic and extraembryonic stem cells without requiring extensive signaling manipulation (Amadei et al., 2021, 2022; Dupont et al., 2023; Harrison et al., 2017; Lau et al., 2022; Sozen et al., 2018; Tarazi et al., 2022; Zhang et al., 2019). Therefore, we focused on generating post-implantation embryoids from EPS cell derivatives. To this end, mouse EPS cells were first induced into PrE/EPI bilineage structures in the microwells of AggreWell plates (Fig. 4C). Separately, TELCs were also generated from mouse EPS cells and seeded into microwells containing the PrE/EPI bilineage structures (Fig. 4C). To promote self-assembly, the culture medium was switched to the basal medium that induces PrE/EPI bilineage structures. From day 3, the embryo-like structures were transferred to 6-well non-adherent suspension culture plates to enhance nutrient supply, and the culture medium was changed to IVC1 medium (Bedzhov et al., 2014). To visualize the self-organization of these EPS-derivatives, PrE/EPI bilineage structures were generated using EPS cells carrying the *Oct4*-GFP reporter, while TELCs were induced from tdTomato-labeled EPS cells.

Next, we monitored the morphological changes during embryoid formation. After 24 h of culture, the cells compacted and formed an aggregate (Fig. 4C). Notably, clumps surrounded by a thin layer of cells efficiently (more than 80%) emerged by day 2 (Fig. 4C), and the *Oct4*-GFP^+^ compartment was segregated from the tdTomato-labeled compartment within the clumps (Fig. S5A). Furthermore, lumen formation was observed in 70.8% of these structures by day 2 (Fig. S5A), and 15% of the cavity-containing structures exhibited both EPI-like and ExE-like lumens (Fig. S5A). After 72 h of culture, the assembled embryoids further elongated into egg-cylinder-like structures, with approximately 60% of these structures still containing cavities. Importantly, 80.91% of the cavity-containing structures displayed a single cavity resembling the pro-amniotic cavity (Fig. S5B and S5C), which forms from the fusion of the embryonic and extra-embryonic cavities during mouse post-implantation development (Bedzhov and Zernicka-Goetz, 2014; Bondarenko et al., 2023; Christodoulou et al., 2018).

We further characterized the egg-cylinder-like embryoids at day 3 using immunofluorescent analysis. In the ExE-like compartment, robust expression of key ExE markers was observed, including CDX2, GATA3, and TFAP2C (Figs. 4D, 4E, S5D and S5E). Meanwhile, the pluripotency marker OCT4 was expressed in the EPI-like compartment (Figs. 4D, 4E, S5D and S5E). Additionally, we detected strong expression of OTX2 in the EPI-like compartment (Fig. S5F), suggesting a successful naïve-to-primed pluripotency transition in this region. In the outer layer, expression of key VE markers, including GATA6, SOX17, and EOMES, was observed (Figs. 4E, 4F and S5G). Notably, consistent with the expression of EOMES and OTX2 in the embryonic part of the VE in natural mouse embryos (Arnold and Robertson, 2009; Kimura et al., 2000; Kwon and Hadjantonakis, 2007), both EOMES and OTX2 were detected in the VE-like layer adjacent to the EPI-like compartment (Figs. 4E and S5F). These findings suggest that EPS-derived cells can self-assemble into egg-cylinder-shaped embryoids, with respect to both morphology and the expression of key marker genes.

At day 5 of culturing, cavities resembling the ectoplacental cavity (EC) and amniotic cavity (AC) were evident and separated by a thin cell layer near the boundary of EPI-like and ExE-like compartment (Fig. 4C), morphologically resembling natural E6.5–E7.0 mouse embryos (Fig. 4G). After an additional day of culture, some embryoids became more complex, resembling natural E7.5 mouse embryos (Fig. 4H). Notably, structures resembling the chorion (Ch) and amnion (Am) emerged (Fig. 4H), along with the formation of exocoelomic cavity (ExC) (Fig. 4H). Taken together, these findings suggest that EPS-derived embryoids can progress to form complex structures that morphologically resemble natural E5.5 to E7.5 mouse embryos.

### EPS cell-derived embryoids recapitulate key developmental events at post-implantation stages

**Figure 5.**
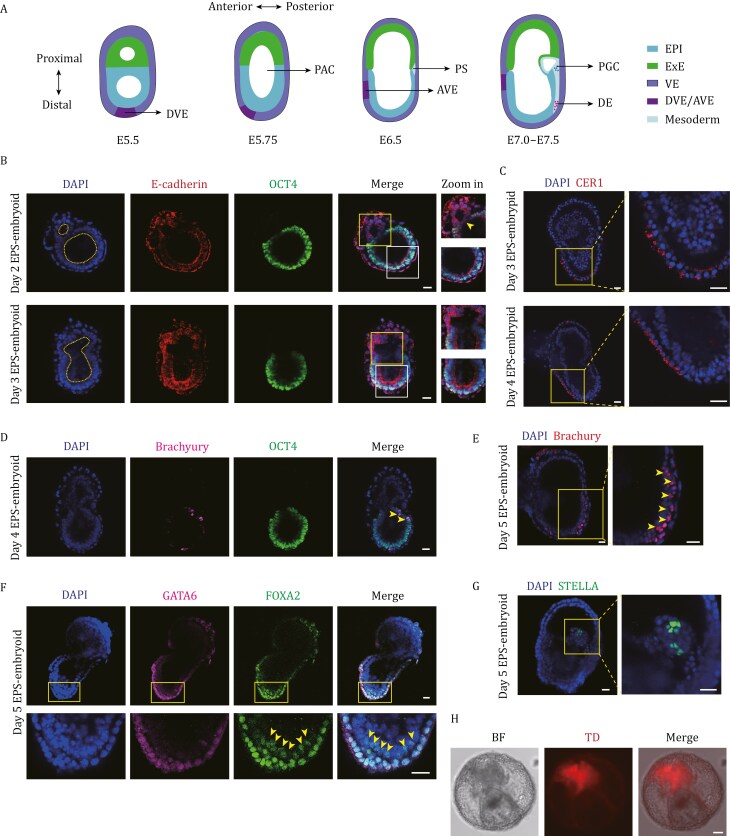
**EPS cell-derived embryoids faithfully model key post-implantation developmental events.** (A) Schematic representation of embryonic development from E5.5 to E7.0/E7.5 *in vivo.* EPI, epiblast; ExE, extraembryonic ectoderm; VE, visceral endoderm; DVE/AVE, distal/anterior visceral endoderm; PAC, pro-amniotic cavity; PS, primitive streak; PGC, primordial germ cells; DE, definitive endoderm. (B) Representative immunofluorescent analysis of E-cadherin (red) and epiblast marker (OCT4, green) expression in (top) Day 2 and (bottom) Day 3 EPS-embryoids. Nuclei were counterstained with DAPI (blue). Yellow boxes indicate the boundary of EPI/ExE-like region, and zoomed fields highlight cavitated areas. Yellow arrowhead indicates cells in the cavitated areas. White boxes show orientation of nuclei in EPI compartments—nuclei become aligned to cavities. Yellow dashed outlines highlight cavities. Scale bar, 25 µm. (C) Representative immunofluorescent analysis showing the expression of CER1 (red) in (top) Day 3 and (bottom) Day 4 EPS-embryoid. Nuclei were counterstained with DAPI (blue). Yellow boxes highlight the magnified regions showing CER1-positive cells. Scale bar, 25 µm. (D) Representative immunofluorescent analysis showing the expression of primitive streak marker Brachyury (magenta) and epiblast marker OCT4 (green) in Day 4 EPS-embryoids. Nuclei were counterstained with DAPI (blue). Scale bar, 25 µm. (E) Representative immunofluorescent analysis showing the expression of primitive streak marker Brachyury (red) and in Day 5 EPS-embryoid. Nuclei were counterstained with DAPI (blue). Yellow box highlights the magnified regions showing Brachyury-positive cells, and yellow arrowheads mark Brachyury-positive cells. Scale bar, 25 µm. (F) Representative immunofluorescent analysis showing the expression of GATA6 (magenta) and FOXA2 (green) in Day 5 EPS-embryoid. Nuclei were counterstained with DAPI (blue). Yellow box highlights the magnified regions showing FOXA2-positive and GATA6-negative cells, and yellow arrowheads mark these cells. Scale bar, 25 µm. (G) Representative immunofluorescent analysis showing the expression of primordial germ cells marker STELLA (green) in Day 5 EPS-embryoid. Nuclei were counterstained with DAPI (blue). Yellow box highlights the magnified regions showing STELLA positive cells. Scale bar, 25 µm. (H) Representative bright-field and tdTomato (TD) fluorescence images of Day 7 EPS-embryoid. Structures show TD expression in chorion-like region. Scale bar, 100 µm.

We next aimed to determine whether EPS cell-derived embryoids could be used to model key developmental events during post-implantation development (Fig. 5A). To this end, we first focused on investigating the formation of the pro-amniotic cavity, which represents a critical morphogenetic event during mouse post-implantation development (Bedzhov and Zernicka-Goetz, 2014; Tam and Loebel, 2007). To visualize cavitation in the EPS cell-derived embryoids, we assessed the expression of E-cadherin, a cell adhesion marker, in these structures between days 2 and 3. On day 2, we observed the formation of two distinct cavities within the EPI-like and ExE-like compartments (Fig. 5B). Notably, the intensity of E-cadherin expression was significantly reduced at the boundary between the EPI-like and ExE-like compartments (Fig. 5B). By day 3, the cavities within the EPI-like and ExE-like compartments had merged into a single large cavity (Fig. 5B). Additionally, we detected the expression of Laminin at the periphery of this large cavity (Fig. S6A), mimicking the formation of a basement membrane-like structure around the boundary of the pro-amniotic cavity in post-implantation mouse embryos.

We further investigated whether the anterior-posterior axis was established in EPS cell-derived embryoids, as this is crucial for the proper induction of the germ layers (Takaoka et al., 2006, 2011; Zernicka-Goetz, 2002). Notably, we found that embryoids at day 3 of development expressed CER1 and LEFTY1, canonical markers for the distal/anterior visceral endoderm (DVE/AVE) (Takaoka et al., 2006) (Figs. 5C and S6B). CER1 and LEFTY1 expression could be detected at the distal tip of the embryoids on day 3 (Figs. 5C and S6B). Moreover, CER1-expressing cells could also be observed asymmetrically on one side of the embryoids (Fig. 5C), suggesting the migration of the AVE from the distal region of the EPI toward the future anterior portion of the embryo.

Having observed robust establishment of the AVE in the anterior region of EPS-derived embryoids, we next investigated whether the primitive streak (PS) formed in the posterior region, marking the initiation of gastrulation. To this end, we examined the expression of Brachyury, a key marker for PS formation. On day 4, Brachyury-expressing cells emerged in the posterior side of the EPI-like compartment, near the ExE-like boundary (Fig. 5D). By day 5, these cells expanded and migrated toward the distal portion of the EPI-like compartment (Fig. 5E). Additionally, we observed the presence of FOXA2^+^GATA6^−^ cells in the distal region of the EPI-like compartment (Fig. 5F), suggesting the formation of the definitive endoderm (DE) during gastrulation. Consistent with this, FOXA2^+^SOX17^+^ cells were also detected in this region (Fig. S6C). Furthermore, a population of FOXA2^+^SOX17^−^ cells was found within the EPI-like compartment (Fig. S6D), implying the formation of the embryo’s midline. On day 5, we also detected STELLA^+^ cells at the boundary between the EPI-like and ExE-like compartments (Fig. 5G), implying the emergence of primordial germ cells (PGCs).

In addition to the development of embryonic compartments, we also sought to analyze the formation of extraembryonic tissues in EPS-derived embryoids. By extending the culture to day 7, the embryo-like structures became enveloped by membranes resembling the amnion (Am) and exhibited an enlarged yolk sac (Fig. 5H). Moreover, we observed chorion-like structures in the embryoids at day 7, derived from tdTomato-labeled TELCs (Fig. 5H). Further immunofluorescent analysis revealed that these chorion-like tissues expressed CK18 (Fig. S6E), a marker for trophoblast lineages. Additionally, we detected cells expressing RUNX1 in the membrane of yolk sac-like tissues (Fig. S6E), suggesting the formation of blood islands in the extraembryonic regions. Collectively, these findings demonstrate that EPS cell-derived embryoids effectively recapitulate key aspects of post-implantation development, including formation of pro-amniotic cavity and AVE, PS induction, gastrulation, and the differentiation of extraembryonic tissues.

### Single cell transcriptomic analysis reveals the similarity between EPS cell-derived embryoids at day 6 and E7.5 natural mouse embryos

**Figure 6.**
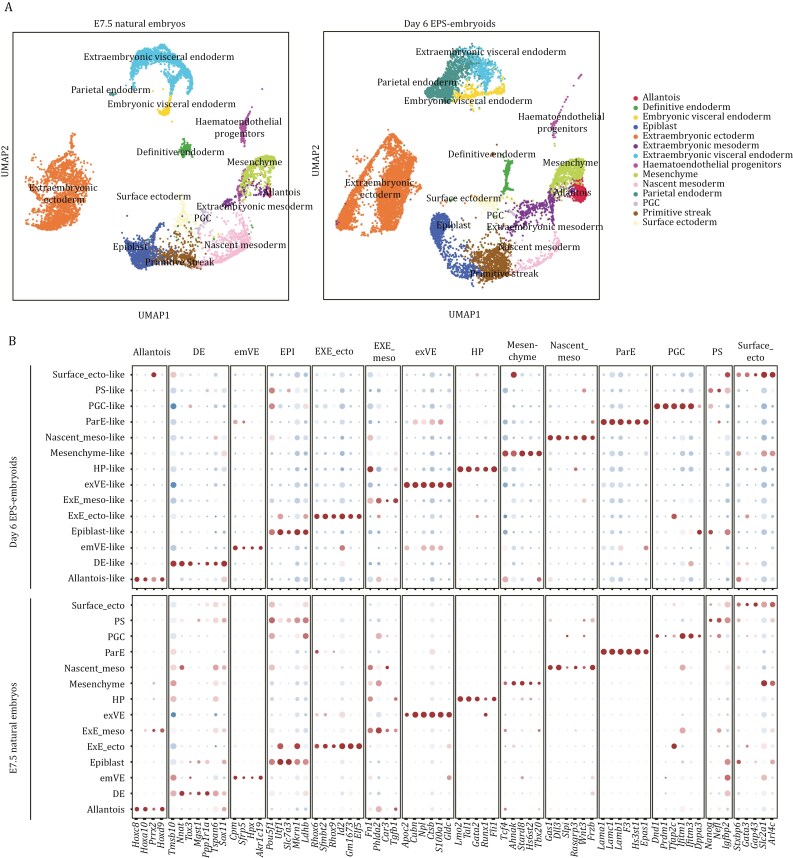
**Day 6 EPS cell-derived embryoids capture major cell types of E7.5 natural embryos.** (A) UMAP of transcriptome of cells from Day 6 EPS-embryoids. These were integrated with published single-cell transcriptomic datasets from E7.5 natural mouse embryos (E-MTAB-6967). (B) Dot plots showing the average levels and proportion of cells expression cell type-specific marker genes in Day 6 EPS-embryoids. Cell type-specific marker genes for different lineages in E7.5 mouse were identified using published single-cell transcriptomic datasets (E-MTAB-6967). Expression of these marker genes in different lineages from E7.5 mouse embryos are shown as the control. DE, definitive endoderm; emVE, Embryonic visceral endoderm; EXE_ecto, extraembryonic ectoderm; EXE_meso, extraembryonic mesoderm; exVE, extraembryonic visceral endoderm; HP, haematoendothelial progenitors; Nascent_meso, nascent mesoderm; PGC, primordial germ cells; ParE, Parietal endoderm; Surface_ecto, surface ectoderm.

To characterize the transcriptomic features of different lineages in EPS cell-derived embryoids at a late stage, we performed single-cell RNA sequencing. Due to the limitations of static culturing conditions, which do not provide sufficient oxygen and nutrients for the organogenesis observed in natural mouse embryos (Fig. S7A), the embryoids at day 6 did not show further advancement in embryonic development. This resulted in the collapse of the embryo-like structures in the embryoids by day 7 (Fig. 5H). Therefore, we used embryoids at day 6 for single-cell RNA sequencing. Notably, UMAP analysis revealed 14 distinct cell clusters in the EPS cell-derived embryoids at day 6 (Fig. 6A), which corresponded to those present in natural E7.5 mouse embryos (Fig. 6A). These cell clusters included lineages from all three germ layers, as well as diverse cell types from extraembryonic tissues (Fig. 6A). Furthermore, Dot plot analysis showed the expression of lineage-specific marker genes in these 14 cell types from the embryoids, mirroring the gene expression profiles observed in natural E7.5 mouse embryos (Fig. 6B).

We further quantified the percentages of each lineage in the total cell population from EPS cell-derived embryoids at day 6. Among the embryonic lineages, the percentages of definitive endoderm, epiblast, primitive streak, and surface ectoderm in the embryoids were comparable to those in natural E7.5 embryos (Fig. S7B). However, the percentage of nascent mesoderm showed a noticeable discrepancy between the embryoids and natural E7.5 embryos (Fig. S7B). Additionally, the total percentage of extraembryonic lineages differed significantly between the embryoids and their *in vivo* counterparts (Fig. S7B). To quantify the transcriptomic similarities between the lineages in the embryoids and E7.5 mouse embryos, we performed correlation analysis. This analysis revealed a high correlation (0.93–0.99) across all cell clusters in the embryoids at day 6 when compared to their natural counterparts (Fig. S7C).

Next, we focused on analyzing extraembryonic tissues using the single-cell transcriptomic data. In natural E7.5 mouse embryos, the allantois emerges from the primitive streak, connecting the embryo proper with the trophoblast compartment, while the amnion forms to protect the developing fetus by providing a fluid-filled environment (Inman and Downs, 2007; Pereira et al., 2011; Roberts et al., 2016). Notably, UMAP analysis revealed the presence of cell clusters expressing the allantois marker genes *Tbx4* and *Hoxa13* in both embryoids and natural E7.5 embryos (Fig. S7D). Additionally, within the mesenchymal cell cluster, we identified a subpopulation expressing several amnion marker genes, including *Acta2*, *Pmp22*, and *Nrp1* (Fig. S7E). To further characterize the trophoblast lineages, we annotated the extraembryonic ectoderm cluster from the UMAP using representative marker genes for different trophoblast lineages (Hadas et al., 2024). This analysis identified subpopulations of trophoblast precursors, chorion, ectoplacental cone, and trophoblast giant cells (Fig. S7F).

Finally, to investigate intercellular communication among embryonic- and extraembryonic-like lineages in day 6 embryoids, we performed CellChat analysis. As a reference, we analyzed single-cell transcriptomic data from natural E7.5 mouse embryos. Similar to natural embryos, the embryoids exhibited complex signaling networks between embryonic and extraembryonic compartments (Fig. S8A). Notably, the overall intensity of inter-lineage communication appeared elevated in embryoids compared to their *in vivo* counterparts (Fig. S8A). To assess the functional relevance of these predicted interactions, we focused on the BMP and WNT signaling pathways, both of which are involved primitive streak formation during early post-implantation development (Camacho-Aguilar et al., 2024; Hadas et al., 2024; Haegel et al., 1995; Huelsken et al., 2000; Kelly et al., 2004). CellChat predicted active BMP and WNT signaling between extraembryonic ectoderm-like and primitive streak-like populations in the embryoids, recapitulating the signaling architecture observed in natural embryos (Fig. S8B and S8C). Similar to natural embryos, pharmacological inhibition of either pathway led to a marked reduction in T expression in embryoids, implying their role in initiating primitive streak-like development (Fig. S8D and S8H).

Taken together, these findings demonstrate that EPS cell-derived embryoids at day 6 exhibit similarities in transcriptome and cell-cell communication patterns to natural E7.5 mouse embryos.

## Discussion

In this study, we developed a stepwise protocol to efficiently induce TELCs from mouse EPS cells using small molecules and cytokines/growth factors. By assembling TELCs with PrE/EPI bilineage structures, we established a transgene-free approach to generate embryoids that resemble mouse embryos at post-implantation stages. Notably, these EPS cell-derived embryoids recapitulated key developmental events during post-implantation, particularly gastrulation. Single-cell transcriptomic analysis further revealed that the EPS cell-derived embryoids at day 6 closely resemble natural E7.5 mouse embryos. These findings highlight the potential of using mouse EPS cells to model post-implantation development in a transgene-free manner.

A major advancement of this study is the successful induction of pre-implantation TE-like cells from mouse EPS cells. By stepwise modulation of signaling pathways associated with TE specification, the pluripotency regulatory network in mouse EPS cells was dramatically downregulated, accompanied by the gradual activation of the TE program (Figs. 1E, 1F and S1A–G). Notably, this stepwise induction protocol enabled robust generation of TELCs, which efficiently differentiated into trophoblast lineages in chimeric experiments (Fig. 2D–G). More importantly, transcriptomic analysis revealed that primary and early-passage TELCs retain the transcriptional features of pre-implantation TE (Figs. 3A, 3B and 3D–F), which distinguishes them from conventional mouse TS cells. These findings suggest that EPS cell-derived TELCs have the potential to be utilized for exploring the mechanisms of TE specification *in vitro*. However, we also observed that the pre-implantation TE features gradually diminished in TELCs at late passages (Fig. 3A–C). Future optimization of culturing conditions for EPS cell-derived TELCs will be necessary to enable long-term expansion of pre-implantation TE-like cells *in vitro*.

In addition to TELC induction, our study also establishes an optimized protocol for generating PrE/EPI bilineage structures from mouse EPS cells. The presence of PrE- and EPI-like cells was confirmed through chimeric contribution assays, immunofluorescence staining, and transcriptomic profiling (Figs. 4B and S3A–I). Notably, RA and CHIR 99021 were utilized to promote PrE differentiation from mouse EPS cells (Figs. 4B and S3A). Interestingly, this same combination has been reported to efficiently induce trophectoderm (TE) from human naïve pluripotent stem cells (Lemke et al., 2024). This divergence likely reflects species-specific differences in the developmental potential of pluripotent stem cells. Human naïve pluripotent stem cells have been shown to possess TE differentiation capacity (Dong and Theunissen, 2022; Dong et al., 2020; Io et al., 2021), whereas mouse naïve pluripotent cells are constrained by an epigenetic barrier that restricts TE specification (Cambuli et al., 2014; Kaiser et al., 2020; Ng et al., 2008). This intrinsic biological distinction may underlie the differential lineage responses to similar inductive signals observed between human and mouse systems.

Another important discovery is that both embryonic and extraembryonic EPS cell-derived derivatives can be assembled to self-organize into post-implantation embryoids in a transgene-free manner. The *in vitro* development of EPS cell-derived embryoids morphologically recapitulated the sequential formation of the pro-amniotic cavity, ectoplacental cavity, amniotic cavity, and exocoelomic cavity (Figs. 4C, 4G, 4H and S5C). Furthermore, immunofluorescent analysis revealed other key developmental events in these embryoids, including AVE formation, PS induction, gastrulation, and the generation of complex extraembryonic tissues (Figs. 5 and S6). Consistent with these observations, single-cell transcriptomic analysis demonstrated the high transcriptional similarity of various embryonic and extraembryonic lineages between EPS cell-derived embryoids at day 6 and natural E7.5 mouse embryos (Fig. S7). These results support the notion that a transgene-free approach can be established to model post-implantation development of mouse embryos with mouse EPS cells, distinguishing it from recent reports that utilize mouse pluripotent stem cells and those overexpressing key extraembryonic regulators (Amadei et al., 2021, 2022; Dupont et al., 2023; Lau et al., 2022; Tarazi et al., 2022). However, it is important to note that the current strategy of assembling EPS derivatives does not recapitulate the native interactions between TE and EPI cells prior to implantation, which may limit the fidelity of the resulting post-implantation embryo model. Future efforts aimed at developing totipotent cell-based approaches may enable more comprehensive *in vitro* modeling of mouse embryogenesis across both pre- and post-implantation stages.

In summary, our study demonstrates the feasibility of generating extraembryonic TE lineages from mouse EPS cells and constructing transgene-free embryoids to model post-implantation development of mouse embryos. These findings underscore the plastic potential of mouse EPS cells to self-assemble complex, post-implantation embryo-like structures *in vitro*. EPS cell-derived embryoids thus offer a powerful tool for investigating the mechanisms underlying mouse post-implantation embryogenesis.

## Supplementary data

Supplementary data is available at *Protein & Cell* online https://doi.org/10.1093/procel/pwaf059.

pwaf059_Supplementary_Materials

## Data Availability

The raw sequence data reported in this paper have been deposited in the Gene Expression Omnibus (GEO) database with accession numbers GSE291068 and GSE291069.

## Code availability

Codes are available from the corresponding authors upon reasonable request.
