# Supplementary material for: Trophectoderm-like cells from EPS cells enable generating EPS cell-derived post-implantation embryoids that complete gastrulation
Source: Protein Cell. 2025 Aug 11;17(2):127–44. doi: 10.1093/procel/pwaf059 (PMC12959774; doi:10.1093/procel/pwaf059)
Supplement: pwaf059_Supplementary_Materials [file pwaf059_supplementary_materials.zip › methods-reference.docx]

**Materials and methods**

**Mice**

The mouse strain B6-Tg (C57BL/6-tdTomato) was bought from Beijing Vitalstar Biotechnology Co., Ltd. Other normal mouse strains ICR and C57BL/6J was purchased from Peking University Health Science Center Department of Laboratory Animal Science. All animal experiments were performed in accordance with the NIH guidelines. All mouse experiments were approved by the Institutional Animal Care and Use Committee of Peking University. The mice were housed in a temperature-controlled room (22 ± 1 ℃) with 40%-60% humidity, under a 12-h light/dark cycle between 06:00 and 08:00.

**Cell culture**

All cell lines were cultured under 20% O_2_ and 5% CO_2_ at 37 ℃. Mouse EPS cells were cultured on feeder layer of mitotically inactivated mouse embryonic fibroblasts (MEFs) in serum-free LCMD medium composed of N2B27 basal medium supplement with recombinant human LIF (10 ng/ml; Novoprotein, C017), CHIR 99021 (3 μM; Selleck, S1263), (S)-(+)-Dimethindene maleate (2 μM; Tocris, 1425) and Minocycline hydrochloride (2 μM; Tocris, 3268). N2B27 basal medium was prepared by including: 1:1 mix of DMEM/F12 (Gibco, 11330032) and Neurobasal (Gibco, 21103049), 1% nonessential amino acids (Gibco, 11140050), 1% GlutaMAX (Gibco, 35050061), 0.5% N2 supplement (Gibco, 17502048), 1% B27 supplement (Gibco, 12587010), and 1% penicillin-streptomycin (Gibco, 15140163). Cells were passaged with 0.05% trypsin-EDTA (Gibco, 25300062) every 3 days. Mouse TSCs were cultured on MEF in serum-containing 1640/serum-TSC medium or serum-free FAXY-TSC medium, and passaged with 0.25% trypsin-EDTA (Gibco, 25200056) every 3-4 days when reached 80% confluency. 1640/serum-TSC medium was as follows: 1640/serum-TSC basal medium composed of RPMI-1640 medium (Gibco, 22400089), 20% ES-qualified FBS (VISTECH, SE200-ES), 1% L-glutamine (Gibco, 25030081), 1% sodium pyruvate (Gibco, 11360070), 1% nonessential amino acids, 1% HEPES (Gibco, 15630080), 0.1 mM 2-mercaotoethanol (Gibco, 21985023), 1% penicillin-streptomycin, supplemented with mouse FGF4 (25 μg/mL; Novoprotein, CR66) and heparin (1 μg/mL; Macklin, H811552). FAXY-TSC medium comprises 1:1 mix of DMEM/F12 and Neurobasal with 0.5% N2 supplement, 1% B27 supplement, 1% L-glutamine, 1-thioglycerol (0.15 μM; Sigma, M6145), human bFGF (12.5 ng/mL; Novoprotein, C046), recombinant Activin A (20 ng/mL; Novoprotein, C687), XAV939 (10 μM; Selleck, S1180), and Y27632 (5 μM; Tocris, 1254).

**Cell lines used in the study**

All EPS cell lines or TSCs cell lines were derived from mouse blastocysts as previously reported. EPS cells constitutively expressing tdTomato (TD-EPS) and EPS cells carrying the Oct4 reporter (OG-EPS) were used in this study.

**Compounds test**

To identify positive modulators for CDX2 activation, mouse EPS cells were dissociated and seeded onto Matrigel-coated 96 well plates in LCDM medium. 6 hours after plating, medium was changed to N2B27 basal medium containing the different concentrations of individual compounds. After 3 days of treatment. cells were fixed and stained for CDX2 and DAPI. Then cells were imaged using fluorescence microscope and the percentages of CDX2 positive cells were analyzed using Image J. The final results were normalized using DMSO control.

**CHIR 99021 concentration test**

EPS cells were dissociated and seeded onto Matrigel-coated wells in LCDM. 6 hours after plating, medium was changed to 1640/serum-TSC basal medium supplemented with 3, 6, 10, 20 μM CHIR 99021 respectively.

**Chemical induction of mouse EPS to TELCs**

A two-step strategy was used to generate EPS-TELCs from EPS. Firstly, EPS cells were dissociated using 0.05% trypsin-EDTA and seeded onto Matrigel-coated plates in TE-S1 medium, consisting of 1640/serum basal medium, plus CHIR 99021 (10 μM), mouse FGF4 (25 ng/mL), heparin (1 μg/mL), GA-017 (10 μg/mL), and A8301 (1 μg/mL; Selleck, S7692). 3 days later, the cell morphology changes to a flat epithelial-like appearance, and pre-TELCs formed.

To get mature TELCs, pre-TELCs were then dissociated using 0.25% trypsin-EDTA and seeded onto feeder cells, and cultured in TE-S2 medium consisting of 1:1 mix of DMEM/F12 and Neurobasal, supplemented with 0.5% N2 supplement, 1% B27 supplement, 1% L-glutamine, 1-thioglycerol, FGF4 (25 μg/mL), heparin (1 μg/mL), recombinant Activin A (20 ng/mL), recombinant human BMP7 (25 μg/mL; Novoprotein, C28B) and 8Br cAMP (1 mg/mL). After 2 days of culture, numerous TSC-like colonies emerged and filled the entire well within 3-4 days. Although for some cell lines that are not prone to differentiation, a few other cell types with EPS similar morphology intermingled at the first passage. TELCs passaged in TE-S2 medium using 0.25% trypsin-EDTA every 3-4 days.

**RNA extraction and RT-qPCR analysis**

Total RNA was extracted using the Trizol Reagent and Direct-zol RNA Kit (ZYMO Research, R2052) according to the manufacturer’s instructions. 1 μg of total RNA was converted to cDNA using TranScript First-Strand cDNA Synthesis SuperMix (TransGen Biotech, AT311). qPCR analysis was conducted using the KAPA SYBR FAST qPCR Kit (KAPA Biosystems, KK4601) with the Bio-Rad CFX Connect Real-Time System. The relative levels of transcript expression of target genes were assessed by the ΔΔCt method. All primers used for qPCR analysis were listed in Table S4.

**Preparation of AggreWell^TM^400 plates**

AggreWell^TM^400 24-well plates (Stem Cell Technologies, 34415) were used in this study and were prepared according to the manufacturer’s instructions. Briefly, each well was pretreated with 500 μL Anti-Adherence Rinsing Solution (Stem Cell Technologies, 07010), centrifuged for 5 min at 2, 000g and incubated at room temperature for 20 min. Next the wells were washed with 2 ml of PBS. After washed, 500 μL modified EPS-blastoid induction medium was added to each well and the plate was centrifuged for 5 min at 2,000g and then placed at 37 ℃ until ready to use.

**Formation of PrE/EPI bilineage structures from mouse EPS cells**

Firstly, EPS cells were dissociated using 0.05% trypsin-EDTA and seeded onto Matrigel-coated plates in PrE induction medium for 3 days, which comprising: modified N2B27 basal medium supplement with mouse FGF4 (50 ng/mL), heparin (1 μg/mL), CHIR 99021 (3 μM), retinotic acid (20 nM; Sigma, R2625) and 8Br cAMP (1 mM; Selleck, S7857). B27 supplement minus insulin (Gibco, A1895601) was used in modified N2B27 basal medium to replace normal B27 supplement. Next, cells were dissociated using 0.25% trypsin-EDTA and seeded at a density of 20, 000 per well on Aggrewell in modified EPS-blastoid induction medium for another 24-36 hours. EPS-blastoid induction medium was as followed: 2:1:1 mix of KSOM medium, N2B27 basal medium and 1640/serum-TS basal medium supplemented with mouse FGF4 (12.5 ng/mL), heparin (0.5 μg/mL), human BMP4 (5 ng/mL; Novoprotein, CR93), CHIR 99021 (3 μM), A8301 (0.5 μM), Y27632 (2 μM) and recombinant Activin A (20 ng/mL). Activin A was supplemented to facilitate the formation of DVE/AVE-like structures(Yamamoto et al., 2004, Kumar et al., 2015). Cells treated with PrE induction medium for 3 days and cell aggregates treated with modified EPS-blastoid induction medium for 36 hours were used for bulk RNA sequencing and further analysis.

**Generation of blastoids *in vitro***

To generate blastoids, 20,000 EPS-TELCs and 20, 000 cells treated with PrE induction medium for 3 days were dissociated. The cell mixture was seeded in EPS-blastoid induction medium within Aggrewell plates for 2 days, enabling efficient blastoid formation. Supplementation of the standard EPS-blastoid induction medium with 10 µM GA-017 significantly enhanced the induction efficiency.

**Generation of EPS-embryoids *in vitro***

Firstly, we induced BLES and EPS-TELCs from EPS as mentioned above, and once the BLES was formed upon 24-36 hours of EPS-blastoid medium treating on Aggrewell (Day 0), EPS-blastoid induction medium was removed completely and EPS-TELCs were dissociated using 0.25% trypsin-EDTA and seeded at a density of 12, 000 cells per well in aggregation medium consisting of 2:1:1 mix of KSOM medium, N2B27 basal medium and TSC condition basal medium. On the following 2 days (day 1-2), 1 mL of medium from each was slowly removed and replaced with 1 ml of fresh medium. Over the course of 2 days without any chemical treating, these cell aggregates self-assembled into an elongated cylindrical architecture typical of post-implantation mouse embryo. On Day 3, 1 mL medium was removed from each well and 1.5 mL same medium was added. And on the same day, EPS-embryoids in the Aggrewell were transferred to suspension cell culture 6-well plate (BEAVER, 40406) for further culture with 4 ml of IVC1 medium (with 20% FBS) per well. From Day 5 to Day 7, well-organized structures were picked every day for further culture in fresh IVC2 (with 30% FBS) medium.

IVC1 and IVC2 medium were as described (Bedzhov et al., 2014). IVC1 medium comprises advanced DMEM/F12 supplement with 20% ES-qualified FBS, 1% GlutaMax, 1% ITS-X (Gibco, 51500056), 1% penicillin-streptomycin, 8 nM β-estradiol (Sigma, E8857), 200 ng/mL progesterone (Sigma, V900699), and 25 μM N-acetyl-L-cysteine (Sigma, A9165). IVC2 medium comprises same components with IVC1 medium except 30% ES-qualified FBS.

**Mouse natural embryos *ex vivo* culture under static conditions**

The embryos were cultured in vitro following a standardized protocol as described (Bedzhov et al., 2014). Briefly, E3.5 embryos were recovered by flushing uteri with M2 medium. Zona pellucida were removed by brief exposure to acidic Tyrode’s solution. Zona-free blastocysts were then seeded in 96-well plates and cultured sequentially in IVC1 and IVC2 (with 30% KSR (Gibco, 10828028)) media. To further support development beyond E6.5, a 1:1 mixture of IVC2 and rat serum was used for an additional 2 days, resulting in the formation of E8.5-like embryos.

**Manipulation of BMP and WNT pathways in natural embryos and EPS-embryoids**

E3.5 natural embryos were collected and sequentially cultured in IVC1 medium for 2 days, followed by an additional 2 days in IVC2 medium supplemented with 30% KSR. On Day 4, embryos displaying proper morphology were selected for chemical treatment in fresh IVC2 medium for 2 days. Day 3 EPS-embryoids with correct morphology were also subjected to drug treatment in IVC medium for 2 days. To manipulate the BMP and WNT pathways, the following reagents were used: BMP4 (100 ng/mL), CHIR99021 (3 µM), IWP2 (5 µM; Selleck, S7085), and LDN-193189-2HCl (2 µM, Selleck, S7507). Each treatment group included at least 8 embryos or embryoids.

**Cell immunofluorescence**

Cells were fixed with 4% paraformaldehyde (DingGuo, AR-0211) at room temperature for 15 min, then rinsed three times in PBS. Permeabilization and blocking were performed with blocking buffer (PBS that contained 3% normal donkey serum (Jackson ImmunoResearch, 017-000-121), and 0.2% Triton X-100 (Sigma-Aldrich, T8787)) at room temperature for 1 h. Then cells were incubated with primary antibodies diluted using blocking buffer at 4 ℃ overnight. After washed 3 times with PBS, cells were incubated with secondary antibodies (Jackson ImmunoResearch) diluted with PBS that contained 3% normal donkey serum at room temperature for 1 h. Finally, the nuclei were stained with DAPI (5 mg/ml; Roche Life Science, 10236276001) at room temperature for 3 min and washed 3 times with PBS. The primary antibodies and secondary antibodies used in this study were listed in Table S5.

**Immunostaining of E4.5 embryos**

E4.5 embryos were washed 3 times with PBS and fixed in 4% paraformaldehyde droplets at room temperature for 20 min. After washed 3 times with PBS, permeabilization and blocking were performed in blocking buffer (as above) at room temperature for 1 h. Primary antibodies were diluted with blocking buffer, and incubated embryos at 4 ℃ overnight. After rinsed 3 times in PBS droplets. Secondary antibodies were diluted with PBS that contained 3% normal donkey serum, and incubated embryos at room temperature for 1 h. Next, the nuclei were stained with DAPI droplets at room temperature for 15 min and washed 3 times with PBS. Finally, embryos were transferred to confocal dish in PBS droplets covered with paraffin for imaging. Confocal microscopy imaging was performed using Leica TCS-SP8. The antibodies used were listed in Table S5.

**Whole-mount immunostaining of post-implantation chimeric embryos and EPS-embryoids**

Whole-mount embryos and EPS-embryoids staining was performed as previously reported (Xu et al., 2022). Briefly, the dissected natural embryos and EPS-embryoids were fixed with 4% paraformaldehyde at room temperature for 20 min and washed three times with PBST (PBS with 0.05% Tween 20). Then permeabilization and were performed with PBS that contained 0.3% Triton-X and 0.1% glycine for 30 min at room temperature. Primary antibody incubation was performed in antibody buffer (PBS contained 10% FBS and 1% Tween 20) at 4 ℃ overnight. After washing 3 times with PBST for 5 min, secondary antibody incubation was performed in antibody buffer (as above) at 4 ℃ overnight followed by another three washes with PBST. Finally, DAPI (5 mg/ml) was added before confocal imaging. For antibody used, see Table S5.

**Chimera assay**

Mouse 8-cell embryos were flushed from ICR females that were super-ovulated by injection of pregnant mare’s serum gonadotropin and human chorionic gonadotropin 2-days after and were mated with ICR males. Embryos were flushed from the oviducts and recovered in M2 medium (Millipore, MR-015-D), then transferred to KSOM-AA and cultured (Millipore, MR-106-D) at 37 ℃ and 5% CO_2_ for follow-up injection. Microinjections were performed

Before injection, PrE induction medium treated cells and EPS-TELCs induced using TD-EPS were dissociated by 0.25-trypsin-EDTA and resuspended using respective induction medium. The cell suspension was placed on ice or 4 ℃ until cell injection. To obtain chimeric blastocysts, 10-15 cells were injected into each 8-cell stage embryo. Injected embryos were transferred into KSOM-AA medium for further development to E4.5, or transferred to uterine horns of 0.5 dpc pseudo-pregnant females and dissected at E6.5. Chimeric embryos of different stage were imaged, fixed and immunostained.

**Bulk RNA-seq data analysis**

**Bulk RNA-seq data processing**

All raw reads were first trimmed by Trimmomatic (version 0.39) software to remove adapters and low-quality reads. Then, cleaned data were mapped to the mouse reference genome (mm10) using STAR (version 2.7.10b) with default parameters. The count matrix of gene expression in each sample was generated by featureCounts (version 2.0.6). The gene expression level was normalized by Transcripts Per Kilobase Million (TPM).

**GO analysis**

GO analysis was performed on DEGs or gene sets using the function enrichGO of the R package ClusterProfiler (version 4.10.0). GO terms with *p* value < 0.05 were defined as significantly enriched.

**Gene Set Variation Analysis (GSVA)**

GSVA was conducted to assess the status of EPS and pre-TELCs using DEGs of E4.5 EPI and E4.5 TE calculated by function FindMarkers of Seurat R package (version 4.3.0.1) using data from GSE123046 (Nowotschin et al., 2019). The gene sets of TE and EPI are listed in Table S3. GSVA was performed with standard settings as implemented in the GSVA R package (1.50.0) in EPS cells, pre-TELCs and cells treated by CHIR99021 with different concentrations. The score was normalized by the max enrichment score. We collected the public data CRR464340 (Liu et al., 2023), extracted the TE structure-like cells, merged them into pseudobulk, performed the same normalization as bulk data, and then used EPI, TE and PrE differential genes (obtained from GSE123046, listed in Table S3) to assess different cell type states.

**Gene Set Enrichment Analysis (GSEA)**

GSEA was performed using DEGs of primary TELCs and FAXY-TS cells with ClusterProfiler software (version 4.10.0). The gene sets of TE and ExE used for GSEA are listed in Table S3 and were calculated by function FindMarkers of Seurat R package using E4.5 TE and E5.5 ExE data from GSE123046.

**Integrated analysis of bulk RNA-seq data and scRNA-seq data**

For the integrated analysis of bulk data (14 samples) and scRNA-seq data (from GSE123046), regarding the co-dimensionality reduction analysis of the two datasets, we first constructed Seurat objects respectively and obtained the variable genes of the two datasets through the standard process. Then, we applied the CCA method implemented in the Seurat package. The maximum number of statistically valid CCA dimensions is constrained by the smaller dataset size minus one (n-1). Since our bulk dataset contained 14 samples, we used the first 13 CCA dimensions (dims = 1:13) for integration. Integration anchors were identified using FindIntegrationAnchors with k.score = 13 to evaluate anchor consistency across all informative dimensions. Batch correction was performed via IntegrateData with k.weight = 13, ensuring appropriate neighborhood size for weight calculation during data harmonization. After integration, we performed co-dimensionality reduction using ScaleData, RunPCA, and RunUMAP with default parameters. For visualization, we extracted the UMAP coordinates of each cell type, and for each single-cell type, we took the median of the UMAP coordinates of all its single cells as the representative point of the type. In an alternative approach (Fig. S2J), we first merged the same types of single-cell data into pseudo-bulk data, then used TPM for normalization and the ComBat function with default parameters in the R package sva (version 3.50.0) to correct batch effects. Then, we performed PCA analysis using the prcomp function based on EPI, TE, and ExE specific genes sets gained from GSE123046 (listed in Table S3). For the clustering analysis of the two datasets, using the pseudo-bulk data, we calculated the Euclidean distance with the expression of EPI, TE and ExE gene sets as mentioned above, and then implemented hierarchical clustering using hclust function with factoextra (version 1.0.7) R package for visualization. The correlation was calculated using the pearson method, and the corrplot package (version 0.92) was used for visualization.

**Single-cell RNA-seq data analysis**

**Single-cell RAN-seq data processing**

The scRNA-seq data were collected and mapped to mouse reference genome (mm10) using Cell Ranger (version 5.0.1) for all our samples. We performed preprocessing using Seurat (version 4.3.0.1). In detail, quality control was firstly performed to remove the cells with (1) total UMI counts < 2000, (2) detected gene number < 1500, or (3) mitochondrial UMI counts > 10%. The normalization was performed using function NormalizeData with default parameters. We performed the standard Seurat clustering pipeline using the following functions: FindVariableFeatures with 2000 genes, ScaleData, RunPCA, FindNeighbors with first 25 PCs, and FindClusters with resolution 1.5. The UMAP dimension reduction was performed with first 25 corrected PCs using function RunUMAP. The DEGs were analyzed using function FindAllMarkers based on normalized gene expression.

**Integration analysis**

For integration analysis, we collected public scRNA seq data of mouse embryos as reference (E-MTAB-6967) (Pijuan-Sala et al., 2019). The preprocess was performed as the same procedures as described above. Following the quality control conditions mentioned in the corresponding article, the CCA (canonical correlation analysis) method in Seurat was used to integrate our data and published data. SelectIntegrationFeatures followed by FindIntegrationAnchors and IntegrateData functions were used to perform integration analysis. Then, the integrated data was used to perform the standard Seurat clustering pipeline as mentioned above. UMAP was later implemented via the RunUMAP function for visualization.

**Correlation analysis**

For correlation analysis of E7.5 embryo in vivo (E-MTAB-6967) and in vitro, using the Seurat package, the average integrated top 2000 variable gene expression data for each cell type was calculated with the AverageExpression function. The correlation was calculated using the pearson method, and the scatter plot using the ggpubr package (version 0.6.0) was used for visualization.

**Cell‒cell communication analysis**

The R package CellChat (version 1.5.0) was employed to analyze cell-to-cell communication between cells in vitro and in vivo. First, a CellChat object was created by grouping defined cell types. The ligandreceptor interaction database we used for analysis was “Secreted Signaling” in CellChatDB.mouse. Preprocessing steps were all conducted with default parameters. The functions computeCommunProb and computeCommunProbPathway were applied to infer the network of each ligand‒receptor pair and each signaling pathway separately. The circle plot with igraph R package (version 1.5.1) was used for visualization. We collected public data GSE169210 as reference (Mittnenzweig et al., 2021).

**References**

BEDZHOV, I., LEUNG, C. Y., BIALECKA, M. & ZERNICKA-GOETZ, M. 2014. In vitro culture of mouse blastocysts beyond the implantation stages. *Nat Protoc,* 9**,** 2732-9.

KUMAR, A., LUALDI, M., LYOZIN, G. T., SHARMA, P., LONCAREK, J., FU, X. Y. & KUEHN, M. R. 2015. Nodal signaling from the visceral endoderm is required to maintain Nodal gene expression in the epiblast and drive DVE/AVE migration. *Dev Biol,* 400**,** 1-9.

LIU, K., XU, X., BAI, D., LI, Y., ZHANG, Y., JIA, Y., GUO, M., HAN, X., LIU, Y., SHENG, Y., KOU, X., ZHAO, Y., YIN, J., LIU, S., CHEN, J., WANG, H., WANG, Y., LIU, W. & GAO, S. 2023. Bilineage embryo-like structure from EPS cells can produce live mice with tetraploid trophectoderm. *Protein Cell,* 14**,** 262-278.

MITTNENZWEIG, M., MAYSHAR, Y., CHENG, S., BEN-YAIR, R., HADAS, R., RAIS, Y., CHOMSKY, E., REINES, N., UZONYI, A., LUMERMAN, L., LIFSHITZ, A., MUKAMEL, Z., ORENBUCH, A. H., TANAY, A. & STELZER, Y. 2021. A single-embryo, single-cell time-resolved model for mouse gastrulation. *Cell,* 184**,** 2825-2842.e22.

NOWOTSCHIN, S., SETTY, M., KUO, Y. Y., LIU, V., GARG, V., SHARMA, R., SIMON, C. S., SAIZ, N., GARDNER, R., BOUTET, S. C., CHURCH, D. M., HOODLESS, P. A., HADJANTONAKIS, A. K. & PE'ER, D. 2019. The emergent landscape of the mouse gut endoderm at single-cell resolution. *Nature,* 569**,** 361-367.

PIJUAN-SALA, B., GRIFFITHS, J. A., GUIBENTIF, C., HISCOCK, T. W., JAWAID, W., CALERO-NIETO, F. J., MULAS, C., IBARRA-SORIA, X., TYSER, R. C. V., HO, D. L. L., REIK, W., SRINIVAS, S., SIMONS, B. D., NICHOLS, J., MARIONI, J. C. & GöTTGENS, B. 2019. A single-cell molecular map of mouse gastrulation and early organogenesis. *Nature,* 566**,** 490-495.

XU, Y., ZHAO, J., REN, Y., WANG, X., LYU, Y., XIE, B., SUN, Y., YUAN, X., LIU, H., YANG, W., FU, Y., YU, Y., LIU, Y., MU, R., LI, C., XU, J. & DENG, H. 2022. Derivation of totipotent-like stem cells with blastocyst-like structure forming potential. *Cell Res,* 32**,** 513-529.

YAMAMOTO, M., SAIJOH, Y., PEREA-GOMEZ, A., SHAWLOT, W., BEHRINGER, R. R., ANG, S. L., HAMADA, H. & MENO, C. 2004. Nodal antagonists regulate formation of the anteroposterior axis of the mouse embryo. *Nature,* 428**,** 387-92.
