# Supplementary material for: Trophectoderm-like cells from EPS cells enable generating EPS cell-derived post-implantation embryoids that complete gastrulation
Source: Protein Cell. 2025 Aug 11;17(2):127–44. doi: 10.1093/procel/pwaf059 (PMC12959774; doi:10.1093/procel/pwaf059)
Supplement: pwaf059_Supplementary_Materials [file pwaf059_supplementary_materials.zip › PAC-25122-XJ-Supplementary file.pdf]

Materials and methods

Mice

The mouse strain B6-Tg (C57BL/6-tdTomato) was bought from Beijing Vitalstar Biotechnology Co., Ltd. Other normal mouse strains ICR and C57BL/6J was purchased from Peking University Health Science Center Department of Laboratory Animal Science. All animal experiments were performed in accordance with the NIH guidelines. All mouse experiments were approved by the Institutional Animal Care and Use Committee of Peking University. The mice were housed in a temperature-controlled room (22 ± 1 °C) with 40%-60% humidity, under a 12-h light/dark cycle between 06:00 and 08:00.

Cell culture

All cell lines were cultured under 20% O_2 and 5% CO_2 at 37 °C. Mouse EPS cells were cultured on feeder layer of mitotically inactivated mouse embryonic fibroblasts (MEFs) in serum-free LCMD medium composed of N2B27 basal medium supplement with recombinant human LIF (10 ng/ml; Novoprotein, C017), CHIR 99021 (3 μM; Selleck, S1263), (S)-(+)-Dimethindene maleate (2 μM; Tocris, 1425) and Minocycline hydrochloride (2 μM; Tocris, 3268). N2B27 basal medium was prepared by including: 1:1 mix of DMEM/F12 (Gibco, 11330032) and Neurobasal (Gibco, 21103049), 1% nonessential amino acids (Gibco, 11140050), 1% GlutaMAX (Gibco, 35050061), 0.5% N2 supplement (Gibco, 17502048), 1% B27 supplement (Gibco, 12587010), and 1% penicillin-streptomycin (Gibco, 15140163). Cells were passaged with 0.05% trypsin-EDTA (Gibco, 25300062) every 3 days. Mouse TSCs were cultured on MEF in serum-containing 1640/serum-TSC medium or serum-free FAXY-TSC medium, and passaged with 0.25% trypsin-EDTA (Gibco, 25200056) every 3-4 days when reached 80% confluency. 1640/serum-TSC medium was as follows: 1640/serum-TSC basal medium composed of RPMI-1640 medium (Gibco, 22400089), 20% ES-qualified FBS (VISTECH, SE200-ES), 1% L-glutamine (Gibco, 25030081), 1% sodium pyruvate (Gibco, 11360070), 1% nonessential amino acids, 1% HEPES (Gibco, 15630080), 0.1 mM 2-mercaotoethanol (Gibco, 21985023), 1% penicillin-streptomycin, supplemented with mouse FGF4 (25 μg/mL; Novoprotein, CR66) and heparin (1 μg/mL; Macklin, H811552). FAXY-TSC medium comprises 1:1 mix of DMEM/F12 and Neurobasal with 0.5% N2 supplement, 1% B27 supplement, 1% L-glutamine, 1-thioglycerol (0.15 μM; Sigma, M6145), human bFGF (12.5 ng/mL; Novoprotein, C046), recombinant Activin A (20 ng/mL; Novoprotein, C687), XAV939 (10 μM; Selleck, S1180), and Y27632 (5 μM; Tocris, 1254).

Cell lines used in the study

All EPS cell lines or TSCs cell lines were derived from mouse blastocysts as previously reported. EPS cells constitutively expressing tdTomato (TD-EPS) and EPS cells carrying the Oct4 reporter (OG-EPS) were used in this study.

Compounds test

To identify positive modulators for CDX2 activation, mouse EPS cells were dissociated and seeded onto Matrigel-coated 96 well plates in LCDM medium. 6 h after plating, medium was changed to N2B27 basal medium containing the different concentrations of individual compounds. After 3 days of treatment. cells were fixed and stained for CDX2 and DAPI. Then

cells were imaged using fluorescence microscope and the percentages of CDX2 positive cells were analyzed using Image J. The final results were normalized using DMSO control.

CHIR 99021 concentration test

EPS cells were dissociated and seeded onto Matrigel-coated wells in LCDM. 6 h after plating, medium was changed to 1640/serum-TSC basal medium supplemented with 3, 6, 10, 20 μM CHIR 99021 respectively.

Chemical induction of mouse EPS to TELCs

A two-step strategy was used to generate EPS-TELCs from EPS. Firstly, EPS cells were dissociated using 0.05% trypsin-EDTA and seeded onto Matrigel-coated plates in TE-S1 medium, consisting of 1640/serum basal medium, plus CHIR 99021 (10 μM), mouse FGF4 (25 ng/mL), heparin (1 μg/mL), GA-017 (10 μg/mL), and A8301 (1 μg/mL; Selleck, S7692). 3 days later, the cell morphology changes to a flat epithelial-like appearance, and pre-TELCs formed. To get mature TELCs, pre-TELCs were then dissociated using 0.25% trypsin-EDTA and seeded onto feeder cells, and cultured in TE-S2 medium consisting of 1:1 mix of DMEM/F12 and Neurobasal, supplemented with 0.5% N2 supplement, 1% B27 supplement, 1% L-glutamine, 1-thioglycerol, FGF4 (25 μg/mL), heparin (1 μg/mL), recombinant Activin A (20 ng/mL), recombinant human BMP7 (25 μg/mL; Novoprotein, C28B) and 8Br cAMP (1 mg/mL). After 2 days of culture, numerous TSC-like colonies emerged and filled the entire well within 3-4 days. Although for some cell lines that are not prone to differentiation, a few other cell types with EPS similar morphology intermingled at the first passage. TELCs passaged in TE-S2 medium using 0.25% trypsin-EDTA every 3-4 days.

RNA extraction and RT-qPCR analysis

Total RNA was extracted using the Trizol Reagent and Direct-zol RNA Kit (ZYMO Research, R2052) according to the manufacturer's instructions. 1 μg of total RNA was converted to cDNA using TranScript First-Strand cDNA Synthesis SuperMix (TransGen Biotech, AT311). qPCR analysis was conducted using the KAPA SYBR FAST qPCR Kit (KAPA Biosystems, KK4601) with the Bio-Rad CFX Connect Real-Time System. The relative levels of transcript expression of target genes were assessed by the ΔΔCt method. All primers used for qPCR analysis were listed in Table S4.

Preparation of AggreWell™400 plates

AggreWell™400 24-well plates (Stem Cell Technologies, 34415) were used in this study and were prepared according to the manufacturer's instructions. Briefly, each well was pretreated with 500 μL Anti-Adherence Rinsing Solution (Stem Cell Technologies, 07010), centrifuged for 5 min at 2, 000g and incubated at room temperature for 20 min. Next the wells were washed with 2 ml of PBS. After washed, 500 μL modified EPS-blastoid induction medium was added to each well and the plate was centrifuged for 5 min at 2,000g and then placed at 37 °C until ready to use.

Formation of PrE/EPI bilineage structures from mouse EPS cells

Firstly, EPS cells were dissociated using 0.05% trypsin-EDTA and seeded onto Matrigel-coated

plates in PrE induction medium for 3 days, which comprising: modified N2B27 basal medium supplement with mouse FGF4 (50 ng/mL), heparin (1 μg/mL), CHIR 99021 (3 μM), retinotic acid (20 nM; Sigma, R2625) and 8Br cAMP (1 mM; Selleck, S7857). B27 supplement minus insulin (Gibco, A1895601) was used in modified N2B27 basal medium to replace normal B27 supplement. Next, cells were dissociated using 0.25% trypsin-EDTA and seeded at a density of 20, 000 per well on Aggrewell in modified EPS-blastoid induction medium for another 24-36 h. EPS-blastoid induction medium was as followed: 2:1:1 mix of KSOM medium, N2B27 basal medium and 1640/serum-TS basal medium supplemented with mouse FGF4 (12.5 ng/mL), heparin (0.5 μg/mL), human BMP4 (5 ng/mL; Novoprotein, CR93), CHIR 99021 (3 μM), A8301 (0.5 μM), Y27632 (2 μM) and recombinant Activin A (20 ng/mL). Activin A was supplemented to facilitate the formation of DVE/AVE-like structures(Yamamoto et al., 2004, Kumar et al., 2015). Cells treated with PrE induction medium for 3 days and cell aggregates treated with modified EPS-blastoid induction medium for 36 h were used for bulk RNA sequencing and further analysis.

Generation of blastoids *in vitro*

To generate blastoids, 20,000 EPS-TELCs and 20, 000 cells treated with PrE induction medium for 3 days were dissociated. The cell mixture was seeded in EPS-blastoid induction medium within Aggrewell plates for 2 days, enabling efficient blastoid formation. Supplementation of the standard EPS-blastoid induction medium with 10 μM GA-017 significantly enhanced the induction efficiency.

Generation of EPS-embryoids *in vitro*

Firstly, we induced BLES and EPS-TELCs from EPS as mentioned above, and once the BLES was formed upon 24-36 h of EPS-blastoid medium treating on Aggrewell (Day 0), EPS-blastoid induction medium was removed completely and EPS-TELCs were dissociated using 0.25% trypsin-EDTA and seeded at a density of 12, 000 cells per well in aggregation medium consisting of 2:1:1 mix of KSOM medium, N2B27 basal medium and TSC condition basal medium. On the following 2 days (day 1-2), 1 mL of medium from each was slowly removed and replaced with 1 ml of fresh medium. Over the course of 2 days without any chemical treating, these cell aggregates self-assembled into an elongated cylindrical architecture typical of post-implantation mouse embryo. On Day 3, 1 mL medium was removed from each well and 1.5 mL same medium was added. And on the same day, EPS-embryoids in the Aggrewell were transferred to suspension cell culture 6-well plate (BEAVER, 40406) for further culture with 4 ml of IVC1 medium (with 20% FBS) per well. From Day 5 to Day 7, well-organized structures were picked every day for further culture in fresh IVC2 (with 30% FBS) medium.

IVC1 and IVC2 medium were as described (Bedzhov et al., 2014). IVC1 medium comprises advanced DMEM/F12 supplement with 20% ES-qualified FBS, 1% GlutaMax, 1% ITS-X (Gibco, 51500056), 1% penicillin-streptomycin, 8 nM β-estradiol (Sigma, E8857), 200 ng/mL progesterone (Sigma, V900699), and 25 μM N-acetyl-L-cysteine (Sigma, A9165). IVC2 medium comprises same components with IVC1 medium except 30% ES-qualified FBS.

Mouse natural embryos *ex vivo* culture under static conditions

The embryos were cultured in vitro following a standardized protocol as described (Bedzhov et al., 2014). Briefly, E3.5 embryos were recovered by flushing uteri with M2 medium. Zona pellucida were removed by brief exposure to acidic Tyrode's solution. Zona-free blastocysts were then seeded in 96-well plates and cultured sequentially in IVC1 and IVC2 (with 30% KSR (Gibco, 10828028)) media. To further support development beyond E6.5, a 1:1 mixture of IVC2 and rat serum was used for an additional 2 days, resulting in the formation of E8.5-like embryos.

Manipulation of BMP and WNT pathways in natural embryos and EPS-embryoids

E3.5 natural embryos were collected and sequentially cultured in IVC1 medium for 2 days, followed by an additional 2 days in IVC2 medium supplemented with 30% KSR. On Day 4, embryos displaying proper morphology were selected for chemical treatment in fresh IVC2 medium for 2 days. Day 3 EPS-embryoids with correct morphology were also subjected to drug treatment in IVC medium for 2 days. To manipulate the BMP and WNT pathways, the following reagents were used: BMP4 (100 ng/mL), CHIR99021 (3 μM), IWP2 (5 μM; Selleck, S7085), and LDN-193189-2HCl (2 μM, Selleck, S7507). Each treatment group included at least 8 embryos or embryoids.

Cell immunofluorescence

Cells were fixed with 4% paraformaldehyde (DingGuo, AR-0211) at room temperature for 15 min, then rinsed three times in PBS. Permeabilization and blocking were performed with blocking buffer (PBS that contained 3% normal donkey serum (Jackson ImmunoResearch, 017-000-121), and 0.2% Triton X-100 (Sigma-Aldrich, T8787)) at room temperature for 1 h. Then cells were incubated with primary antibodies diluted using blocking buffer at 4 °C overnight. After washed 3 times with PBS, cells were incubated with secondary antibodies (Jackson ImmunoResearch) diluted with PBS that contained 3% normal donkey serum at room temperature for 1 h. Finally, the nuclei were stained with DAPI (5 mg/ml; Roche Life Science, 10236276001) at room temperature for 3 min and washed 3 times with PBS. The primary antibodies and secondary antibodies used in this study were listed in Table S5.

Immunostaining of E4.5 embryos

E4.5 embryos were washed 3 times with PBS and fixed in 4% paraformaldehyde droplets at room temperature for 20 min. After washed 3 times with PBS, permeabilization and blocking were performed in blocking buffer (as above) at room temperature for 1 h. Primary antibodies were diluted with blocking buffer, and incubated embryos at 4 °C overnight. After rinsed 3 times in PBS droplets. Secondary antibodies were diluted with PBS that contained 3% normal donkey serum, and incubated embryos at room temperature for 1 h. Next, the nuclei were stained with DAPI droplets at room temperature for 15 min and washed 3 times with PBS. Finally, embryos were transferred to confocal dish in PBS droplets covered with paraffin for imaging. Confocal microscopy imaging was performed using Leica TCS-SP8. The antibodies used were listed in Table S5.

Whole-mount immunostaining of post-implantation chimeric embryos and EPS-embryoids

Whole-mount embryos and EPS-embryoids staining was performed as previously reported (Xu

et al., 2022). Briefly, the dissected natural embryos and EPS-embryoids were fixed with 4% paraformaldehyde at room temperature for 20 min and washed three times with PBST (PBS with 0.05% Tween 20). Then permeabilization and were performed with PBS that contained 0.3% Triton-X and 0.1% glycine for 30 min at room temperature. Primary antibody incubation was performed in antibody buffer (PBS contained 10% FBS and 1% Tween 20) at 4 ℃ overnight. After washing 3 times with PBST for 5 min, secondary antibody incubation was performed in antibody buffer (as above) at 4 ℃ overnight followed by another three washes with PBST. Finally, DAPI (5 mg/ml) was added before confocal imaging. For antibody used, see Table S5.

Chimera assay

Mouse 8-cell embryos were flushed from ICR females that were super-ovulated by injection of pregnant mare's serum gonadotropin and human chorionic gonadotropin 2-days after and were mated with ICR males. Embryos were flushed from the oviducts and recovered in M2 medium (Millipore, MR-015-D), then transferred to KSOM-AA and cultured (Millipore, MR-106-D) at 37 ℃ and 5% CO_2 for follow-up injection. Microinjections were performed

Before injection, PrE induction medium treated cells and EPS-TELCs induced using TD-EPS were dissociated by 0.25-trypsin-EDTA and resuspended using respective induction medium. The cell suspension was placed on ice or 4 ℃ until cell injection. To obtain chimeric blastocysts, 10-15 cells were injected into each 8-cell stage embryo. Injected embryos were transferred into KSOM-AA medium for further development to E4.5, or transferred to uterine horns of 0.5 dpc pseudo-pregnant females and dissected at E6.5. Chimeric embryos of different stage were imaged, fixed and immunostained.

Bulk RNA-seq data analysis

Bulk RNA-seq data processing

All raw reads were first trimmed by Trimmomatic (version 0.39) software to remove adapters and low-quality reads. Then, cleaned data were mapped to the mouse reference genome (mm10) using STAR (version 2.7.10b) with default parameters. The count matrix of gene expression in each sample was generated by featureCounts (version 2.0.6). The gene expression level was normalized by Transcripts Per Kilobase Million (TPM).

GO analysis

GO analysis was performed on DEGs or gene sets using the function enrichGO of the R package ClusterProfiler (version 4.10.0). GO terms with p value < 0.05 were defined as significantly enriched.

Gene Set Variation Analysis (GSVA)

GSVA was conducted to assess the status of EPS and pre-TELCs using DEGs of E4.5 EPI and E4.5 TE calculated by function FindMarkers of Seurat R package (version 4.3.0.1) using data from GSE123046 (Nowotschin et al., 2019). The gene sets of TE and EPI are listed in Table S3. GSVA was performed with standard settings as implemented in the GSVA R package (1.50.0) in EPS cells, pre-TELCs and cells treated by CHIR99021 with different concentrations. The score was normalized by the max enrichment score. We collected the public data CRR464340

(Liu et al., 2023a), extracted the TE structure-like cells, merged them into pseudobulk, performed the same normalization as bulk data, and then used EPI, TE and PrE differential genes (obtained from GSE123046, listed in Table S3) to assess different cell type states.

Gene Set Enrichment Analysis (GSEA)

GSEA was performed using DEGs of primary TELCs and FAXY-TS cells with ClusterProfiler software (version 4.10.0). The gene sets of TE and ExE used for GSEA are listed in Table S3 and were calculated by function FindMarkers of Seurat R package using E4.5 TE and E5.5 ExE data from GSE123046.

Integrated analysis of bulk RNA-seq data and scRNA-seq data

For the integrated analysis of bulk data (14 samples) and scRNA-seq data (from GSE123046), regarding the co-dimensionality reduction analysis of the two datasets, we first constructed Seurat objects respectively and obtained the variable genes of the two datasets through the standard process. Then, we applied the CCA method implemented in the Seurat package. The maximum number of statistically valid CCA dimensions is constrained by the smaller dataset size minus one (n-1). Since our bulk dataset contained 14 samples, we used the first 13 CCA dimensions (dims = 1:13) for integration. Integration anchors were identified using FindIntegrationAnchors with k.score = 13 to evaluate anchor consistency across all informative dimensions. Batch correction was performed via IntegrateData with k.weight = 13, ensuring appropriate neighborhood size for weight calculation during data harmonization. After integration, we performed co-dimensionality reduction using ScaleData, RunPCA, and RunUMAP with default parameters. For visualization, we extracted the UMAP coordinates of each cell type, and for each single-cell type, we took the median of the UMAP coordinates of all its single cells as the representative point of the type. In an alternative approach (Fig. S2J), we first merged the same types of single-cell data into pseudo-bulk data, then used TPM for normalization and the ComBat function with default parameters in the R package sva (version 3.50.0) to correct batch effects. Then, we performed PCA analysis using the prcomp function based on EPI, TE, and ExE specific genes sets gained from GSE123046 (listed in Table S3). For the clustering analysis of the two datasets, using the pseudo-bulk data, we calculated the Euclidean distance with the expression of EPI, TE and ExE gene sets as mentioned above, and then implemented hierarchical clustering using hclust function with factoextra (version 1.0.7) R package for visualization. The correlation was calculated using the pearson method, and the corrplot package (version 0.92) was used for visualization.

Single-cell RNA-seq data analysis

Single-cell RAN-seq data processing

The scRNA-seq data were collected and mapped to mouse reference genome (mm10) using Cell Ranger (version 5.0.1) for all our samples. We performed preprocessing using Seurat (version 4.3.0.1). In detail, quality control was firstly performed to remove the cells with (1) total UMI counts < 2000, (2) detected gene number < 1500, or (3) mitochondrial UMI counts > 10%. The normalization was performed using function NormalizeData with default parameters. We performed the standard Seurat clustering pipeline using the following functions: FindVariableFeatures with 2000 genes, ScaleData, RunPCA, FindNeighbors with first 25 PCs,

and FindClusters with resolution 1.5. The UMAP dimension reduction was performed with first 25 corrected PCs using function RunUMAP. The DEGs were analyzed using function FindAllMarkers based on normalized gene expression.

Integration analysis

For integration analysis, we collected public scRNA seq data of mouse embryos as reference (E-MTAB-6967) (Pijuan-Sala et al., 2019). The preprocess was performed as the same procedures as described above. Following the quality control conditions mentioned in the corresponding article, the CCA (canonical correlation analysis) method in Seurat was used to integrate our data and published data. SelectIntegrationFeatures followed by FindIntegrationAnchors and IntegrateData functions were used to perform integration analysis. Then, the integrated data was used to perform the standard Seurat clustering pipeline as mentioned above. UMAP was later implemented via the RunUMAP function for visualization.

Correlation analysis

For correlation analysis of E7.5 embryo in vivo (E-MTAB-6967) and in vitro, using the Seurat package, the average integrated top 2000 variable gene expression data for each cell type was calculated with the AverageExpression function. The correlation was calculated using the pearson method, and the scatter plot using the ggpubr package (version 0.6.0) was used for visualization.

Cell–cell communication analysis

The R package CellChat (version 1.5.0) was employed to analyze cell-to-cell communication between cells in vitro and in vivo. First, a CellChat object was created by grouping defined cell types. The ligandreceptor interaction database we used for analysis was “Secreted Signaling” in CellChatDB.mouse. Preprocessing steps were all conducted with default parameters. The functions computeCommunProb and computeCommunProbPathway were applied to infer the network of each ligand–receptor pair and each signaling pathway separately. The circle plot with igraph R package (version 1.5.1) was used for visualization. We collected public data GSE169210 as reference (Mittnenzweig et al., 2021).

Supplementary Figure Legends

Figure S1. High concentration of CHIR 99021 significantly activates *Cdx2* expression in mouse EPS cells.

(A) Representative immunofluorescence images of CDX2 (red) in cells treated with CHIR 99021 (10 µM), FGF4 (100 ng/ml), bFGF (100 ng/ml). Nuclei were counterstained with DAPI (blue). Scale bar, 100 µm.

(B-C) Q-PCR analysis of trophectoderm lineage and pluripotency genes in indicated treating conditions. C3/C6/C10/C20, EPS cells treated with 3/6/10/20 µM CHIR 99021 for 3 days, respectively; TE-S1, EPS cells treated with TE-S1 medium for 3 days. Error bar represents SD of the mean of biological replicates (n=3). Statistical significance was determined by one-way ANOVA. *** $p < 0.001$, ****$p < 0.0001$, ns indicates $p > 0.05$.

(D) Volcano plots depicting the differentially expressed genes (DEGs) between mouse EPS cells and cells treated with 3, 6, 10, 20 µM CHIR 99021, respectively. Upregulated (fold change > 2) and downregulated (fold change < 0.5) genes are shown in red and blue dots. Adjusted *p*-value < 0.05.

(E) Concentration-dependent volcano plots of DEGs between mouse EPS cells and cells treated with increasing concentrations of CHIR 99021.

(F) Representative immunofluorescence images of trophectoderm marker (CDX2, magenta) and pluripotency markers (NANOG, green) in EPS cells and cells treated with TE-S1 medium for 3 days. Nuclei were counterstained with DAPI (blue). Scale bars, 100 µm.

(G) Volcano plots depicting the differentially expressed genes (DEGs) between mouse EPS cells and pre-TELCs (TE-S1). Upregulated (fold change > 2) and downregulated (fold change < 0.5) genes are shown in red and blue dots. Adjusted *p*-value < 0.05.

Figure S2. TELCs exhibit complete exit from pluripotency and robust activation of key trophectoderm markers.

(A) Representative immunofluorescence images of trophectoderm marker (CDX2, magenta) and pluripotency markers (OCT4/Nanog, green) in primary TELCs. Nuclei were counterstained with DAPI (blue). Yellow arrowheads mark OCT4 positive cells. Scale bars, 500 µm.

(B) Q-PCR analysis of trophectoderm lineage genes in cells under various conditions. C10/C20, EPS cells treated with 10/20 µM CHIR 99021 for 3 days, respectively; TELCs (P1/P5/P13), TELCs cultured under TE-S2 conditions at passages 1, 5, and 13. Error bar represents SD of the mean of biological replicates (n=3). Statistical significance was determined by one-way ANOVA (compared with FAXY-TS cells). *p-value < 0.05, **$p < 0.01$, ***$p < 0.001$, ****$p < 0.0001$, ns indicates $p > 0.05$.

(C-E) Representative immunofluorescence images of trophectoderm and pluripotency markers in pre-TELCs and TELCs. Nuclei were counterstained with DAPI (blue). Scale bar, 25 µm.

(F) Representative immunofluorescence images of primary TELCs stained for trophectoderm markers CDX2 (magenta) and EOMES (magenta), mesoderm marker Brachyury (green), and primitive endoderm lineage marker SOX17 (green). Nuclei were counterstained with DAPI (blue). Scale bar, 100 µm.

(G) Heatmap depicting relative expression of epiblast (EPI), trophectoderm (TE), primitive endoderm (PrE) and mesoderm-specific genes across mouse EPS cells, primary TELCs (this study), and TE-like structures from EPS-blastoids (Blastoid TE-like, published data

CRR464340).

(H) Heatmap showing standardized enrichment scores for EPI, TE and PrE-specific gene sets in the cell types analyzed in (G). Top color bar denotes mouse embryonic data, and bottom bar indicates *in vitro* samples..

(I) (Left) Heatmap of cell-state-specific gene expression in EPS cells, pre-TELCs, and TELCs. (Right) Corresponding enriched GO terms.

(J) PCA analysis of bulk RNA-seq data from mouse EPS cells, FAXY-TS cells, pre-TELCs, and TELCs across passages (P1, P3, P6, P21), integrated with pseudobulk scRNA-seq data generated from published mouse embryo datasets (GSE123046), encompassing epiblast and trophoblast lineages across pre-implantation to post-implantation stages, using defined gene sets (n = 1,581 genes covering EPI, TE, and ExE). See Methods for details.

Figure S3. Efficient derivation of bilineage structures with functional EPI and PrE cells from mouse EPS cells.

(A) Q-PCR analysis of (left) PrE lineage genes and (right) pluripotency genes in cells treated with PrE induction medium compared to mouse EPS cells. Error bar represents SD of the mean of biological replicates (n=3). Statistical significance was determined by Student's *t*-test. $**p < 0.01$, $****p < 0.0001$, ns indicates $p > 0.05$.

(B) Representative image showing the contribution of tdTomato (TD) labelled mouse EPS cells treated with PrE induction medium to the EPI (yellow dashed outline) and VE (white dashed outline) regions of the E6.5 chimeric embryo. Scale bar, 25 μm.

(C) Table summarizing the outcomes of E6.5 chimeric embryos assays of cells treated with PrE induction medium for 3 days.

(D) Representative bright-field image of PrE/EPI bilineage structure (BLES). Scale bar, 25 μm.

(E) Representative immunofluorescent analysis showing the expression of GATA6 (magenta) and OCT4 (green) in PrE/EPI bilineage structure (BLES). Nuclei were counterstained with DAPI (blue). Scale bar, 25 μm.

(F-G) Scatterplots displaying the transcriptome change of (F) mouse EPS cell treated with PrE induction medium (OG-PrE induction), and (G) PrE/EPI bilineage structures (OG-BLES) compare with EPS cells (OG-EPS). Upregulated (fold change > 2) and downregulated (fold change < 0.5) genes are shown in red and blue dots, respectively. Adjusted *p*-value < 0.05.

(H) PCA of OG-EPS cells, cells treated with PrE induction medium for 3 days (OG-PrE induction), and PrE/EPI bilineage structures (OG-BLES) based on the global transcriptome.

(I) Heatmap showing the relative expression of pre-implantation and post-implantation EPI and PrE lineage specific genes in cells treated with PrE induction medium (OG-PrE induction) and EPI/PrE bilineage structures (OG-BLES). Naïve and primed pluripotent genes indicate pre- and post- implantation EPI genes.

Figure S4. Generation of blastocyst-like structures from EPS-derived cells.

(A) Schematic diagram of the protocol for generating blastoids from mouse EPS-derived TELCs and EPS cells treated with PrE induction medium.

(B) Representative morphology of blastoids induced from EPS-derived cells. Scale bar, 25 μm.

(C) Representative composite images showing blastoids formed by reaggregation of tdTomato-labeled (TD) TELCs and *Oct4*-GFP (OG) reporter-expressing EPS cells treated with PrE

induction medium: left panel displays bright-field morphology, while the right panel shows corresponding TD and OG fluorescence channels. Scale bar, 500 μm.

(D) Representative images showing decidualization in the mouse uterus 4 days after transferring blastoids at embryonic day 2.5 (E2.5). Scale bars, 5 mm.

Figure S5. Egg-cylinder embryoids self-assembled from EPS-derived cells exhibit accurate morphology and gene expression.

(A-B) Characterization of (A) Day 2 and (B) Day 3 EPS-embryoid. (Left) Representative bright field, tdTomato (TD) and Oct4-GFP (OG) fluorescence images of EPS-embryoids. Scale bar, 25 μm. (Right) Frequency of different types of egg cylinder-like structures.

(C) Representative immunofluorescent image showing the location of OCT4 (green) and tdTomato (TD) positive cells in Day 3 EPS-embryoid. Nuclei were counterstained with DAPI (blue). Scale bar, 25 μm.

(D-G) Representative immunofluorescent analysis showing the expression of epiblast markers (OCT4, OTX2), extraembryonic ectoderm markers (TFAP2C, GATA3), and visceral endoderm VE marker (SOX17) in Day 3 EPS-embryoids. Scale bar, 25 μm.

Figure S6. EPS cell-derived embryoids recapitulate key post-implantation developmental events.

(A) Representative immunofluorescent analysis showing the expression of Laminin (green) in Day 3 EPS-embryoid. Nuclei were counterstained with DAPI (blue). Scale bar, 25 μm.

(B) Representative immunofluorescent analysis showing the expression of LEFTY1 (magenta) and OCT4 (green) in Day 3 EPS-embryoid. Nuclei were counterstained with DAPI (blue). Yellow box highlights the magnified regions showing LEFTY1 positive cells, and yellow arrowheads marks these cells. Scale bar, 25 μm.

(C-D) Representative immunofluorescent analysis showing the expression of SOX17 (magenta) and FOXA2 (Green) in Day 5 EPS-embryoid. Nuclei were counterstained with DAPI (blue). Yellow box highlights the magnified regions showing (C) FOXA2 and Sox17 double positive cells, and (E) FOXA2-positive and SOX17-negative cells, and yellow arrowheads marks these cells, respectively. Scale bar, 25 μm.

(E) Representative immunofluorescent analysis showing the expression of hematopoietic progenitor marker RUNX1 (magenta) and chorion marker CK18 (green) in dissected yolk sac-like membrane from Day 7 EPS-embryoid. Yellow box highlights the magnified regions showing RUNX1 positive cells and CK18 positive cells. Yellow arrowheads mark RUNX1-positive cells and white arrowheads indicate CK18-positive cells. Scale bar, 25 μm.

Figure S7. Day 6 EPS-embryoids exhibit cell types highly similar to E7.5 natural embryos.

(A) Representative bright-field image of mouse E8.5 embryo cultured *ex vivo* under static condition. Scale bar, 100 μm.

(B) The proportion abundance of cell types in natural E7.5 embryo and Day 6 EPS-embryoids.

(C) Correlation of gene expression for each identified lineage between Day 6 EPS-embryoids and natural E7.5 embryos. Correlation coefficients are indicated. DE, definitive endoderm; emVE, Embryonic visceral endoderm; exVE, extraembryonic visceral endoderm; HP, haematoendothelial progenitors; Nascent_meso, nascent mesoderm; PGC, primordial germ

cells; ParE, Parietal endoderm.

(D) Gene expression of the allantois markers *Tbx4* and *Hoxa13* in natural embryos and Day 6 EPS-embryoids.

(E) Gene expression of the selected amnion marker *Acta2*, *Hand1*, *Pmp22*, *Nrp1* and *Foxf1* in natural embryos and Day 6 EPS-embryoids.

(F) Expression of selected markers of (top row) chorion progenitors, (second row) chorion, (third row) ectoplacental cone (EPC), and (bottom row) trophoblast giant cells. For EPC, co-expression of *Hand1* and *Ascl2* indicates uncommitted EPC, and co-expression of *Ascl2* and *Chsy1* indicates committed EPC cells.

Figure S8. Formation of primitive streak requires BMP and WNT signaling.

(A) Comparison of signaling networks between Day 6 EPS-embryoids (this study) and E7.5 natural embryos (published data GSE169210). Circle sizes are proportional to the number of cells in each subpopulation, and line weight indicates interaction probability.

(B-C) Extraembryonic ectoderm-derived signaling outputs for (B) BMP and (C) WNT pathways in Day 6 EPS-embryoids (this study) versus E7.5 natural embryos (published data GSE169210). Line weight denotes signaling probability directed to targets tissues.

(D) Representative immunofluorescence images showing expression of primitive streak marker Brachyury (magenta) and epiblast marker OCT4 (green) in *ex vivo* cultured embryo (top) versus E6.75 natural embryo (bottom). Nuclei were counterstained with DAPI (blue). Scale bar, 50 µm.

(E-H) Representative immunofluorescence images showing expression of primitive streak marker Brachyury (magenta) and epiblast marker OCT4 (green) in *ex vivo* cultured embryos (top) and EPS-embryoids (bottom) after 2-day differential treatments. Nuclei were counterstained with DAPI (blue). Scale bar, 50 µm.

Figure S1

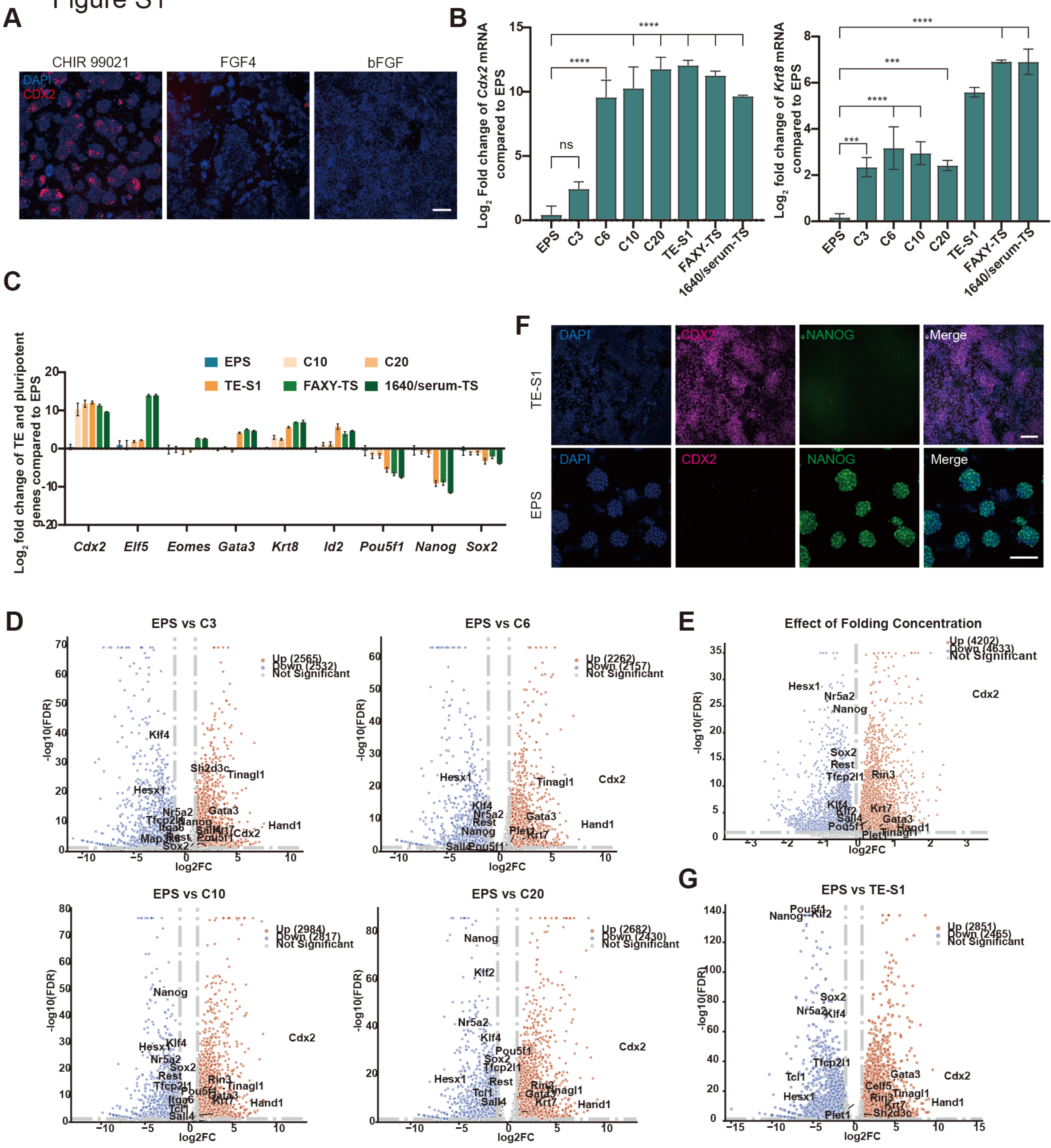
A
CHIR 99021
FGF4
bFGF
DAPI
CDX2
B
Log2 fold change of Cdx2 mRNA compared to EPS
Log2 fold change of Krt8 mRNA compared to EPS
EPS
C3
C6
C10
C20
TE-S1
FAXY-TS
1640/serum-TS
ns
****
***
C
Log2 fold change of TE and pluripotent genes compared to EPS
EPS
C10
C20
TE-S1
FAXY-TS
1640/serum-TS
Cdx2
Elf5
Eomes
Gata3
Krt8
Id2
Pou5f1
Nanog
Sox2
F
TE-S1
EPS
DAPI
CDX2
NANOG
Merge
D
EPS vs C3
EPS vs C6
EPS vs C10
EPS vs C20
-log10(FDR)
log2FC
Up (2565)
Down (2532)
Not Significant
Up (2262)
Down (2157)
Up (2984)
Down (2817)
Up (2682)
Down (2430)
E
Effect of Folding Concentration
Up (4202)
Down (4633)
G
EPS vs TE-S1
Up (2851)
Down (2465)
Cdx2
Hand1
Tinagl1
Gata3
Krt7
Nanog
Klf4
Hesx1
Sox2
Nr5a2
Rest
Tfcp2l1
Sall4
Rin3
Pou5f1
Klf2
Plet1
Tcl1
Sh2d3c
Celf5
Itga6

Figure S2

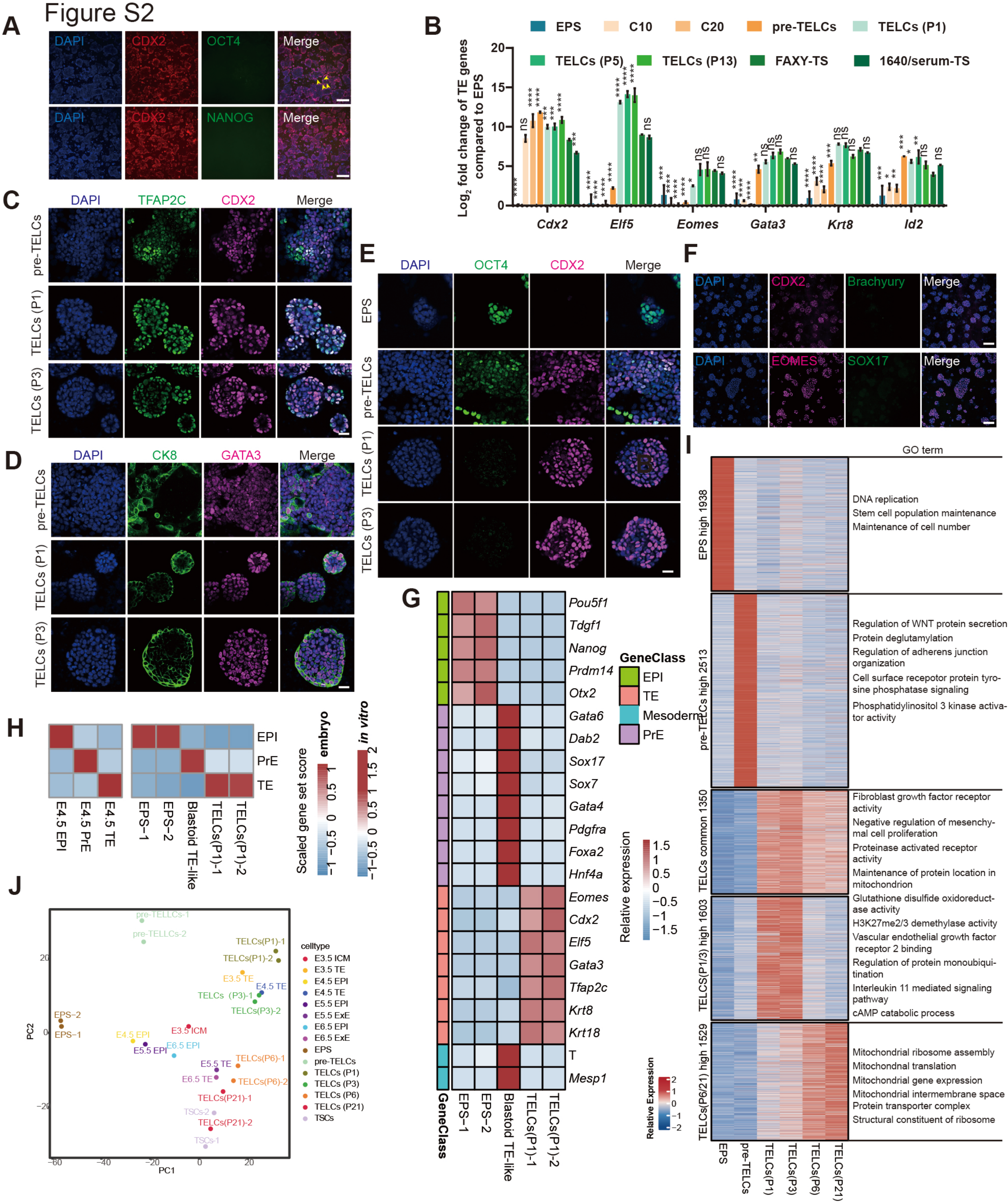
A
DAPI
CDX2
OCT4
Merge
NANOG
B
EPS
C10
C20
pre-TELCs
TELCs (P1)
TELCs (P5)
TELCs (P13)
FAXY-TS
1640/serum-TS
Log2 fold change of TE genes compared to EPS
Cdx2
Elf5
Eomes
Gata3
Krt8
Id2
C
TFAP2C
pre-TELCs
TELCs (P1)
TELCs (P3)
D
CK8
GATA3
E
EPS
F
Brachyury
EOMES
SOX17
G
Pou5f1
Tdgf1
Nanog
Prdm14
Otx2
Gata6
Dab2
Sox17
Sox7
Gata4
Pdgfra
Foxa2
Hnf4a
Eomes
Cdx2
Elf5
Gata3
Tfap2c
Krt8
Krt18
T
Mesp1
GeneClass
EPI
TE
Mesoderm
PrE
Relative expression
EPS-1
EPS-2
Blastoid TE-like
TELCs(P1)-1
TELCs(P1)-2
H
E4.5 EPI
E4.5 PrE
E4.5 TE
Scaled gene set score
embryo
in vitro
I
GO term
EPS high 1938
DNA replication
Stem cell population maintenance
Maintenance of cell number
pre-TELCs high 2513
Regulation of WNT protein secretion
Protein deglutamylation
Regulation of adherens junction organization
Cell surface recepotor protein tyrosine phosphatase signaling
Phosphatidylinositol 3 kinase activator activity
TELCs common 1350
Fibroblast growth factor receptor activity
Negative regulation of mesenchymal cell proliferation
Proteinase activated receptor activity
Maintenance of protein location in mitochondrion
TELCS(P1/3) high 1603
Glutathione disulfide oxidoreductase activity
H3K27me2/3 demethylase activity
Vascular endothelial growth factor receptor 2 binding
Regulation of protein monoubiquitination
Interleukin 11 mediated signaling pathway
cAMP catabolic process
TELCs(P6/21) high 1529
Mitochondrial ribosome assembly
Mitochondrial translation
Mitochondrial gene expression
Mitochondrial intermembrane space
Protein transporter complex
Structural constituent of ribosome
Relative Expression
TELCs(P6)
TELCs(P21)
J
celltype
E3.5 ICM
E3.5 TE
E4.5 EPI
E4.5 TE
E5.5 EPI
E5.5 ExE
E6.5 EPI
E6.5 ExE
EPS
pre-TELCs
TELCs (P1)
TELCs (P3)
TELCs (P6)
TELCs (P21)
TSCs
PC1
PC2

Figure S3

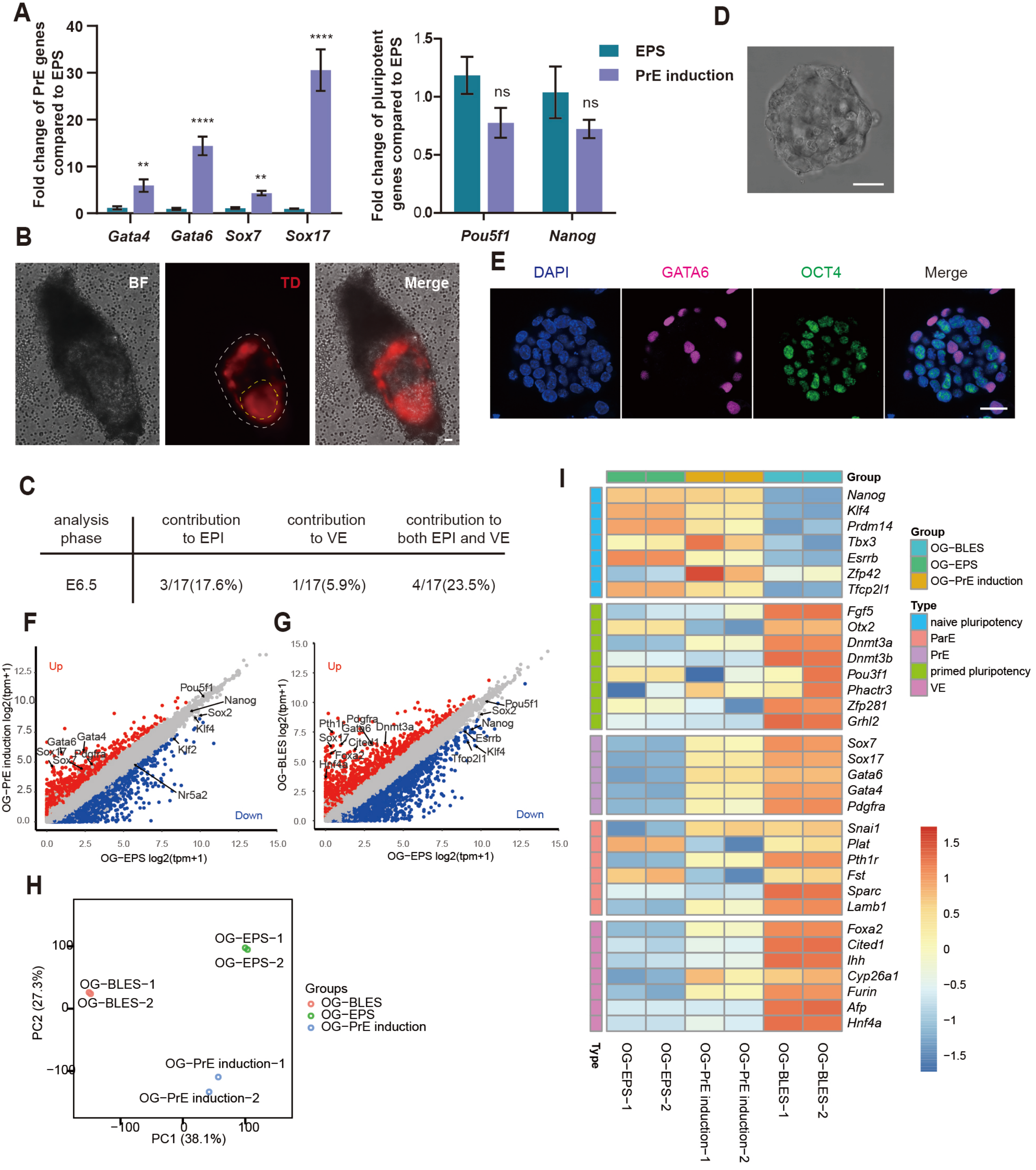

A
Fold change of PrE genes compared to EPS
Gata4
Gata6
Sox7
Sox17
**
****
**
****
Fold change of pluripotent genes compared to EPS
Pou5f1
Nanog
ns
ns
EPS
PrE induction
D
B
BF
TD
Merge
E
DAPI
GATA6
OCT4
Merge
C
analysis phase | contribution to EPI | contribution to VE | contribution to both EPI and VE
E6.5 | 3/17(17.6%) | 1/17(5.9%) | 4/17(23.5%)
F
Up
Down
OG−PrE induction log2(tpm+1)
OG−EPS log2(tpm+1)
Pou5f1
Nanog
Sox2
Klf4
Klf2
Nr5a2
Gata6
Gata4
Sox17
Sox7
Pdgfra
G
Up
Down
OG−BLES log2(tpm+1)
OG−EPS log2(tpm+1)
Pth1r
Pdgfra
Gata6
Dnmt3a
Sox17
Cited1
Foxa2
Hnf4a
Pou5f1
Sox2
Nanog
Esrrb
Klf4
Tfcp2l1
H
PC1 (38.1%)
PC2 (27.3%)
OG−EPS−1
OG−EPS−2
OG−BLES−1
OG−BLES−2
OG−PrE induction−1
OG−PrE induction−2
Groups
OG−BLES
OG−EPS
OG−PrE induction
I
Group
Nanog
Klf4
Prdm14
Tbx3
Esrrb
Zfp42
Tfcp2l1
Fgf5
Otx2
Dnmt3a
Dnmt3b
Pou3f1
Phactr3
Zfp281
Grhl2
Sox7
Sox17
Gata6
Gata4
Pdgfra
Snai1
Plat
Pth1r
Fst
Sparc
Lamb1
Foxa2
Cited1
Ihh
Cyp26a1
Furin
Afp
Hnf4a
Group
OG−BLES
OG−EPS
OG−PrE induction
Type
naive pluripotency
ParE
PrE
primed pluripotency
VE
Type
OG−EPS−1
OG−EPS−2
OG−PrE induction−1
OG−PrE induction−2
OG−BLES−1
OG−BLES−2

Figure S4

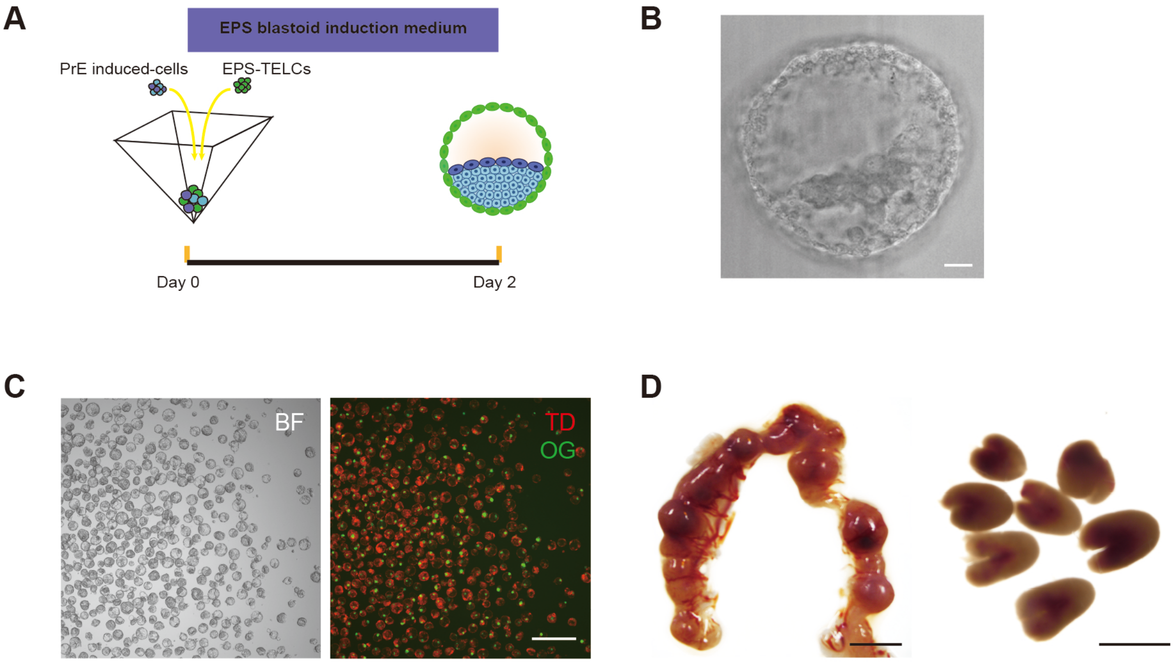
A
EPS blastoid induction medium
PrE induced-cells
EPS-TELCs
Day 0
Day 2
B
C
BF
TD
OG
D

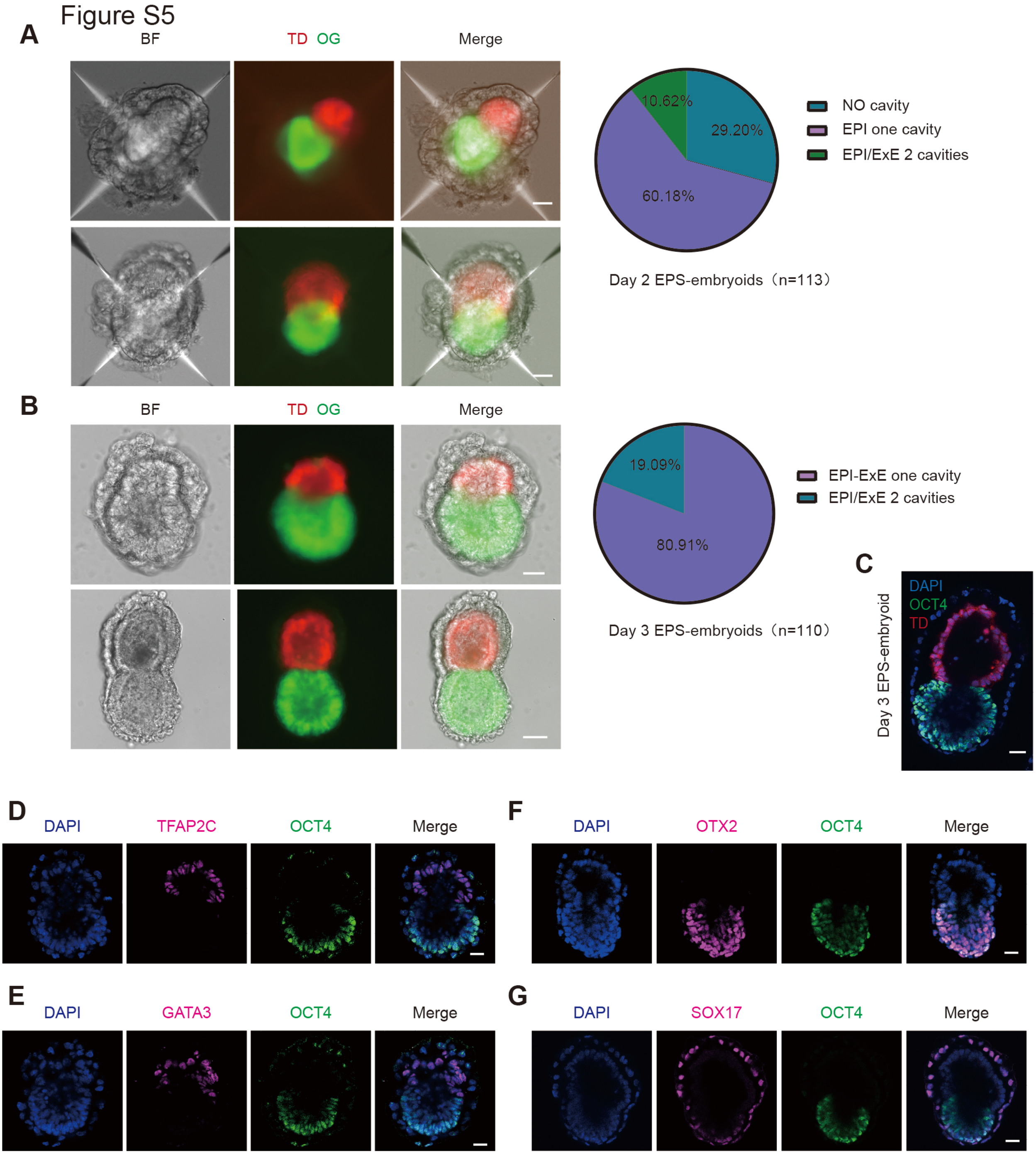
Figure S5
A
BF
TD OG
Merge
10.62%
29.20%
60.18%
NO cavity
EPI one cavity
EPI/ExE 2 cavities
Day 2 EPS-embryoids（n=113）
B
BF
TD OG
Merge
19.09%
80.91%
EPI-ExE one cavity
EPI/ExE 2 cavities
Day 3 EPS-embryoids（n=110）
C
Day 3 EPS-embryoid
DAPI
OCT4
TD
D
DAPI
TFAP2C
OCT4
Merge
F
DAPI
OTX2
OCT4
Merge
E
DAPI
GATA3
OCT4
Merge
G
DAPI
SOX17
OCT4
Merge

Figure S6

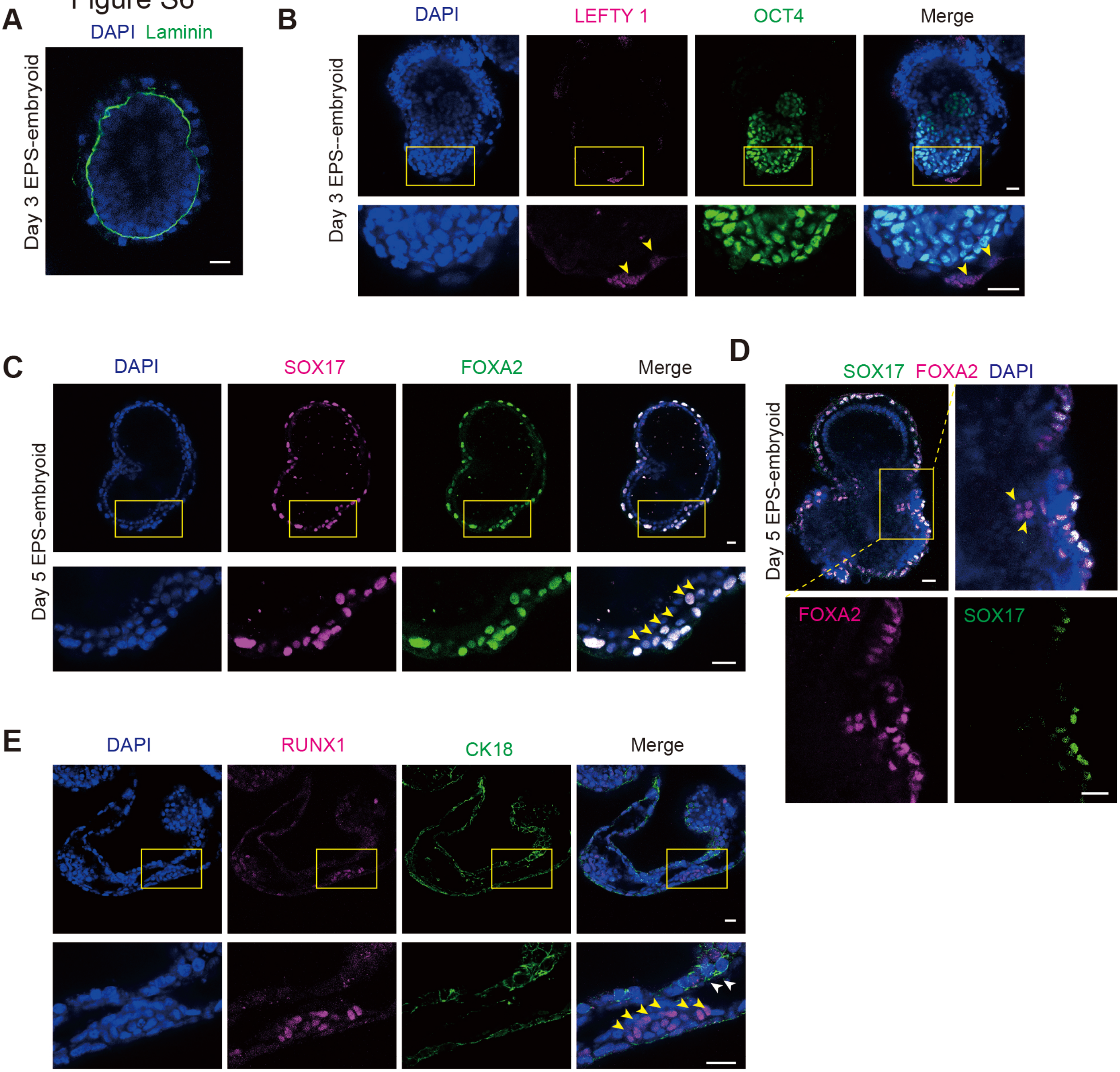

A
DAPI Laminin
Day 3 EPS-embryoid
B
DAPI
LEFTY 1
OCT4
Merge
Day 3 EPS--embryoid
C
DAPI
SOX17
FOXA2
Merge
Day 5 EPS-embryoid
D
SOX17 FOXA2 DAPI
Day 5 EPS-embryoid
FOXA2
SOX17
E
DAPI
RUNX1
CK18
Merge

Figure S7

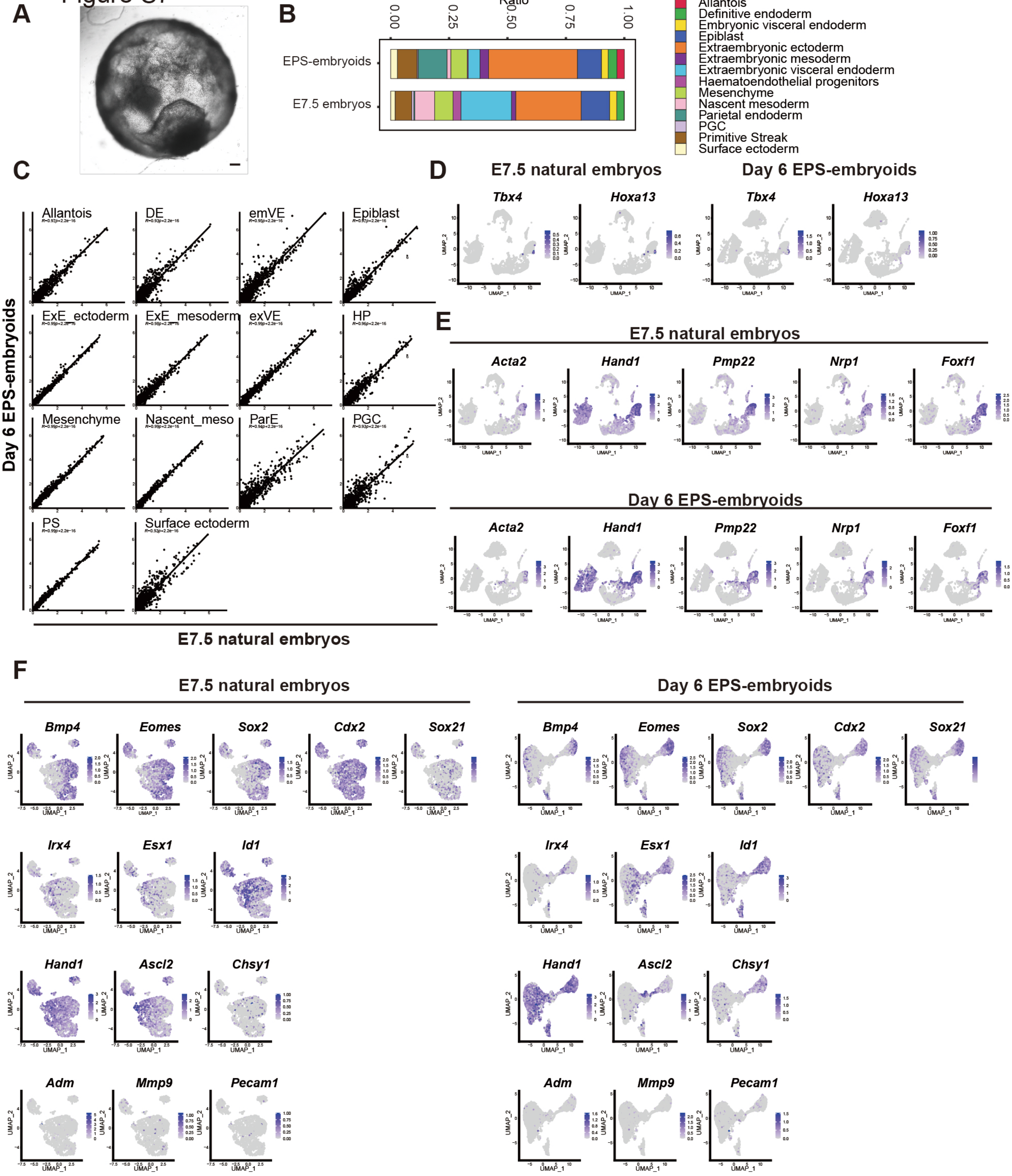
A
B
Ratio
0.00
0.25
0.50
0.75
1.00
EPS-embryoids
E7.5 embryos
celltype
Allantois
Definitive endoderm
Embryonic visceral endoderm
Epiblast
Extraembryonic ectoderm
Extraembryonic mesoderm
Extraembryonic visceral endoderm
Haematoendothelial progenitors
Mesenchyme
Nascent mesoderm
Parietal endoderm
PGC
Primitive Streak
Surface ectoderm
C
Day 6 EPS-embryoids
Allantois
DE
emVE
Epiblast
ExE_ectoderm
ExE_mesoderm
exVE
HP
Mesenchyme
Nascent_meso
ParE
PGC
PS
Surface ectoderm
E7.5 natural embryos
D
E7.5 natural embryos
Day 6 EPS-embryoids
Tbx4
Hoxa13
E
E7.5 natural embryos
Acta2
Hand1
Pmp22
Nrp1
Foxf1
Day 6 EPS-embryoids
F
E7.5 natural embryos
Day 6 EPS-embryoids
Bmp4
Eomes
Sox2
Cdx2
Sox21
Irx4
Esx1
Id1
Hand1
Ascl2
Chsy1
Adm
Mmp9
Pecam1
UMAP_1
UMAP_2

Figure S8

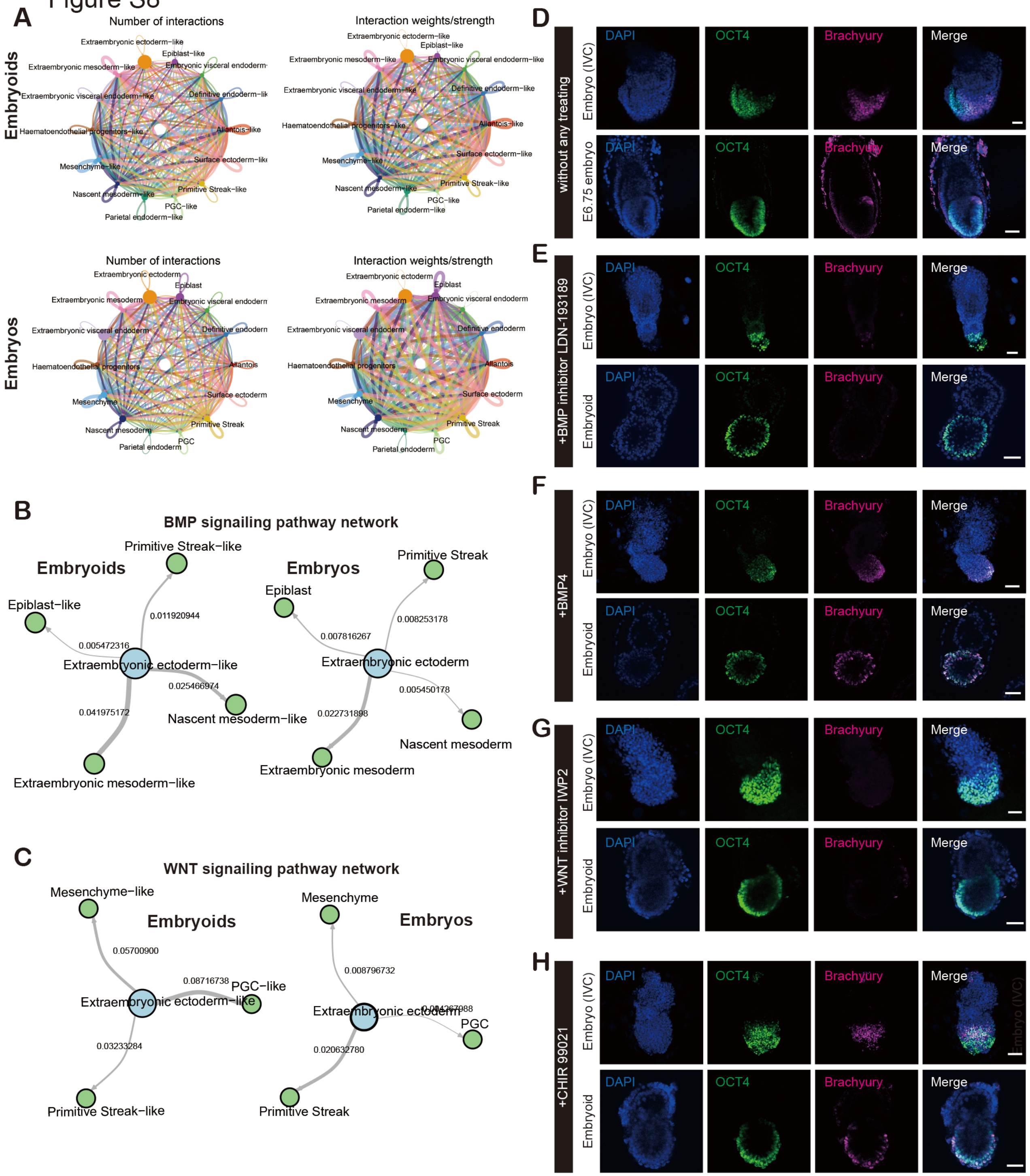
A
Embryoids
Number of interactions
Interaction weights/strength
Extraembryonic ectoderm−like
Epiblast−like
Extraembryonic mesoderm−like
Embryonic visceral endoderm−like
Extraembryonic visceral endoderm−like
Definitive endoderm−like
Haematoendothelial progenitors−like
Allantois−like
Surface ectoderm−like
Mesenchyme−like
Primitive Streak−like
Nascent mesoderm−like
PGC−like
Parietal endoderm−like
Embryos
Extraembryonic ectoderm
Epiblast
Extraembryonic mesoderm
Embryonic visceral endoderm
Extraembryonic visceral endoderm
Definitive endoderm
Haematoendothelial progenitors
Allantois
Surface ectoderm
Mesenchyme
Primitive Streak
Nascent mesoderm
PGC
Parietal endoderm
B
BMP signailing pathway network
Embryoids
Primitive Streak−like
Epiblast−like
0.011920944
0.005472316
Extraembryonic ectoderm−like
0.025466974
Nascent mesoderm−like
0.041975172
Extraembryonic mesoderm−like
Embryos
Primitive Streak
Epiblast
0.008253178
0.007816267
Extraembryonic ectoderm
0.005450178
0.022731898
Nascent mesoderm
Extraembryonic mesoderm
C
WNT signailing pathway network
Mesenchyme−like
Embryoids
0.05700900
0.08716738
PGC−like
Extraembryonic ectoderm−like
0.03233284
Primitive Streak−like
Mesenchyme
Embryos
0.008796732
Extraembryonic ectoderm
0.004267988
PGC
0.020632780
Primitive Streak
D
without any treating
Embryo (IVC)
E6.75 embryo
DAPI
OCT4
Brachyury
Merge
E
+BMP inhibitor LDN-193189
Embryo (IVC)
Embryoid
F
+BMP4
G
+WNT inhibitor IWP2
H
+CHIR 99021
